# Molecular phylogeny, pathogenicity and toxigenicity of *Fusarium oxysporum* f. sp. *lycopersici*

**DOI:** 10.1038/srep21367

**Published:** 2016-02-17

**Authors:** D. Nirmaladevi, M. Venkataramana, Rakesh K. Srivastava, S. R. Uppalapati, Vijai Kumar Gupta, T. Yli-Mattila, K. M. Clement Tsui, C. Srinivas, S. R. Niranjana, Nayaka S. Chandra

**Affiliations:** 1 Department of Microbiology and Biotechnology, Jnanabarathi Campus, Bangalore University, Bangalore, Karnataka, India; 2 DRDO-BU-CLS, Barathiar University Campus, Coimbatore, Tamil Nadu, India; 3 International Crops Research Institute for the Semi-Arid Tropics (ICRISAT), Hyderabad, India; 4 Defence Food Research Laboratory, Siddarthanagar, Mysore, Karnataka, India; 5 Molecular Glycobiotechnology Group, Discipline of Biochemistry, School of Natural Sciences, National University of Ireland, Galway, Ireland; 6 Molecular Plant Biology, Department of Biochemistry, University of Turku, FI-20014 Turku, Finland; 7 Department of Pathology and Laboratory Medicine, The University of British Columbia, Canada; 8 DOS in Biotechnology, University of Mysore, Manasagangothri, Mysore, Karnataka, India

## Abstract

The present study aimed at the molecular characterization of pathogenic and non pathogenic *F. oxysporum* f. sp. *lycopersici* strains isolated from tomato. The causal agent isolated from symptomatic plants and soil samples was identified based on morphological and molecular analyses. Pathogenicity testing of 69 strains on five susceptible tomato varieties showed 45% of the strains were highly virulent and 30% were moderately virulent. Molecular analysis based on the fingerprints obtained through ISSR indicated the presence of wide genetic diversity among the strains. Phylogenetic analysis based on ITS sequences showed the presence of at least four evolutionary lineages of the pathogen. The clustering of *F. oxysporum* with non pathogenic isolates and with the members of other formae speciales indicated polyphyletic origin of *F. oxysporum* f. sp. *lycopersici*. Further analysis revealed intraspecies variability and nucleotide insertions or deletions in the ITS region among the strains in the study and the observed variations were found to be clade specific. The high genetic diversity in the pathogen population demands for development of effective resistance breeding programs in tomato. Among the pathogenic strains tested, toxigenic strains harbored the *Fum1* gene clearly indicating that the strains infecting tomato crops have the potential to produce Fumonisin.

In India, crops are grown under varied agro-climatic conditions and *Fusarium* spp., occur regularly causing considerable crop losses and produce several mycotoxins^1^,^2^. The genetic variability of the members of genus *Fusarium* increases the difficulties encountered in the development of resistant host genotypes, as well as in effectively deploying available tolerant cultivars. *Fusarium oxysporum* is a species complex comprising ubiquitous soil borne plant pathogens with more than 150 host-specific forms or formae speciales^3^,^4^.

Tomato (*Lycopersicon esculentum* Mill.) is one of the world’s most widely cultivated vegetable crops for consumption as fresh fruits and various types of processed products^5^,^6^. Low yield of tomato is attributed to its susceptibility to several pathogenic fungi, bacteria, viruses and nematodes which are major constraints to tomato cultivation^7^. Fusarium wilt caused by the soil borne fungus, *Fusarium oxysporum* Schlectend.: Fr. f. sp. *lycopersici* (Sacc.) W.C. Snyder and H.N. Hansen, is one of the most devastating diseases of tomato^8^. It affects greenhouse and field grown tomatoes in warm vegetable production areas. The disease is characterized by yellowed leaves and wilted plants with minimal or absent crop yield. There may be a 30 to 40% yield loss due to the disease and this may go up to 80% under favorable weather conditions^9^,^10^. High Fusarium wilt incidence in tomato of 25–55% has been recorded from various parts of India^11^,^12^. The pathogen invades the root epidermis and extends into the vascular tissue. It colonizes the xylem vessels producing mycelium and conidia. The characteristic wilt symptoms appear as a result of severe water stress, mainly due to vessel clogging^13^. Three physiological races (1, 2, and 3) of the pathogen are distinguished by their specific pathogenicity to tomato cultivars^14^. Since *F. oxysporum* f. sp. *lycopersici* (Fol) is an asexual fungus, genetic exchange occurs via somatic fusion and hetreokaryon formation between vegetative compatible strains^15^.

Chemical treatments and soil solarisation in the fields usually fail to control the pathogen. Use of resistant cultivars is the most reliable method of disease prevention. The selection of an appropriate cultivar requires a thorough understanding of the form and race of the pathogen emerging in the field^16^. Genetic diversity and phylogenetic analyses within local populations of the pathogen help in elucidating the emergence and evolutionary relationships between the pathogenic and non-pathogenic strains and also may provide information of the pathogen dispersal from other geographical areas^17^,^18^. A detailed understanding of the population structure and diversity within Fol is also crucial for the development of effective disease management strategies^19^. Determining the level of genetic variability within pathogen populations is important since a high genetic variation indicates rapid evolution in response to the changing environmental conditions and the development of new races overcoming host resistance^20^,^21^,^22^.

A variety of approaches had been employed for the characterization of *F. oxysporum* f. sp. *lycopersici* isolates. Elias *et al.*^23^ studied the population structure of Fol based on RFLP analysis of the genomic DNA which provided genetic evidence that Vegetative Compatibility Grouping (VCG) was an indicator of evolutionary origin. Based on Vegetative Compatibility, mtDNA RFLP, IGS and isozyme polymorphism, several researchers showed that isolates of Fol might represent two genetically distinct evolutionary lineages^24^,^25^,^26^,^27^. Hirano and Arie^28^, compared the endopolygalacturonase (*pg1)* and exopolygalacturonase (*pgx4*) gene sequences of Fol and Forl from Japan for their differentiation based on PCR. Phylogenetic analyses of sequences of the elongation factor α (EF-1α) and *pgx4* gene were conducted by Lievens *et al.*^29^ on strains of Fol. Mishra *et al.*^30^ determined diversity among Fol isolates based on RAPD. Phoutthasone *et al.*^31^ used virulence and AFLP markers for the genetic differentiation between Fol populations of Thailand.

Mycotoxins are the secondary metabolites produced by certain molds on a wide range of agricultural commodities and are closely related to human and animal food chains^32^,^33^. Fumonisins are mycotoxins produced by at least 11 species of the fungus *Fusarium*, including plant pathogens^34^,^35^. Fumonisin produced by *Fusarium* spp., is one of the important toxins which has become a serious constraint in major food crops during the last two decades^36^. The wide range and frequent presence of Fumonisin toxin found naturally occurring on crops reveal an increasing need for research on the toxigenic potential of *Fusarium* spp. affecting plants other than cereals to assess the extent of mycotoxin hazard to man and animals.

In view of the economic importance of *Fusarium* wilt of tomato, the objectives of this study were to investigate phylogenetic relationships between *F. oxysporum* f. sp. *lycopersici* and non pathogenic *F. oxysporum* strains isolated from different tomato growing regions of South India through molecular markers, gene sequence analysis, virulence analysis on different tomato varieties as well as the detection of toxigenic strains.

## Results

### Morphological identification

All the isolates displayed morphology typical to *Fusarium oxysporum*. The mycelia of *Fusarium oxysporum* isolates appeared delicate, white to pink, often with purple tinge, and were sparse to abundant. The fungus produced three types of spores: macroconidia, microconidia and chlamydospores. Macroconidia, sparse to abundant, are borne on branched conidiophores or on the surface of sporodochia and are thin walled, three- to five-septate, fusoid-subulate and pointed at both ends, have pedicellate base. The macroconidia usually measured 15–37.5 μ × 2.5–4 μ and the three-septate macroconidia were more common. Microconidia were abundant, borne on simple phialides arising laterally, oval-ellipsoid, straight to curved, 2.5–15 μ × 2–3 μ usually nonseptate or single septate. Chlamydospores, both smooth and rough walled, were abundant and formed terminally or on an intercalary basis. They are generally solitary, but occasionally form in pairs or chains. The *Fusarium oxysporum* isolates exhibited a high level of diversity in terms of culture and morphology.

### Species specific PCR assay

**Figure 1:**
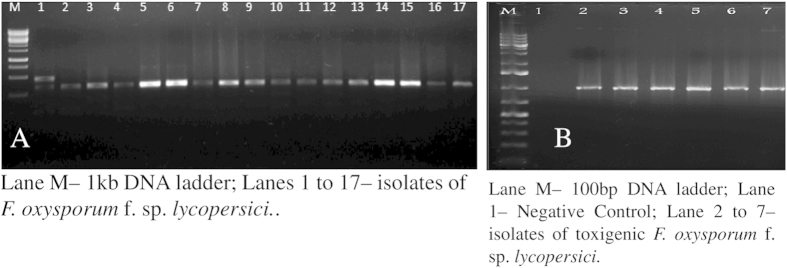
Detection of *F. oxysporum* species and its fumonisin genotypes by PCR assay. (**A**) Species-specific PCR assay. (**B**) Detection of fumonisin positive *F. oxysporum* species.

Species specific PCR assay was used for the specific detection of *F. oxysporum*. PCR with species specific primers amplified a single 340 bp DNA fragment specific to *F. oxysporum*, thus confirming the species specific identification (Fig. 1A).

### Pathogenicity assay

Typical symptoms of wilt disease were first observed 15–20 days after inoculation. In the virulence test, variation in symptoms on aerial parts and within the stem tissues of tomato plants infected with Fol was observed. At early stage, symptoms appeared as yellowing of the lower leaves and in later stages, drooping of the leaves was observed. In severe infection, the pith of the stem was turned brown in colour. In severely infected plants lower leaves dried, ultimately the aerial parts of the tomato plant showed loss of turgidity and drooped down. The results of pathogenicity tests carried out on five cultivars of tomato with 69 isolates of *F. oxysporum* are presented in Table 1. The *F. oxysporum* isolates examined had differences in the disease severity on test tomato varieties. Based on the mean disease severity (MDS), the virulence of each isolate was recorded as low (MDS: <25%), moderate (MDS: 25–50%) or high (MDS: >50%). The isolates were thus categorized into 4 groups viz., Highly pathogenic (31 strains), Moderately pathogenic (20 strains), Weakly pathogenic (12 strains) and Non-pathogenic (6 strains) based on the symptomatological variations in the test tomato varieties. The uninoculated tomato seedlings showed no symptoms. Based on the Root-dip inoculation test isolates pathogenic to tomato were identified as *F. oxysporum* f. sp. *lycopersici.* All Fol isolates were successfully reisolated from disease-affected plants, thereby completing Koch’s postulates.

### ISSR fingerprinting

**Figure 2:**
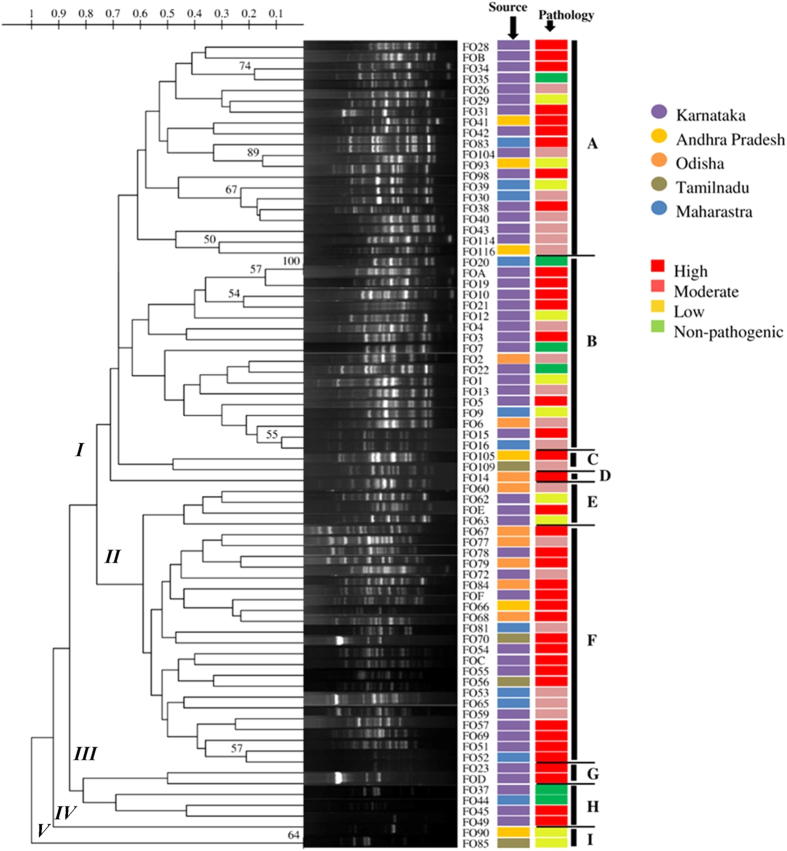
Phylogenetic analysis *F. oxysporum* f. sp. *lycopersici* isolates by ISSR analysis with Primer (GA)_9_T.

**Figure 3:**
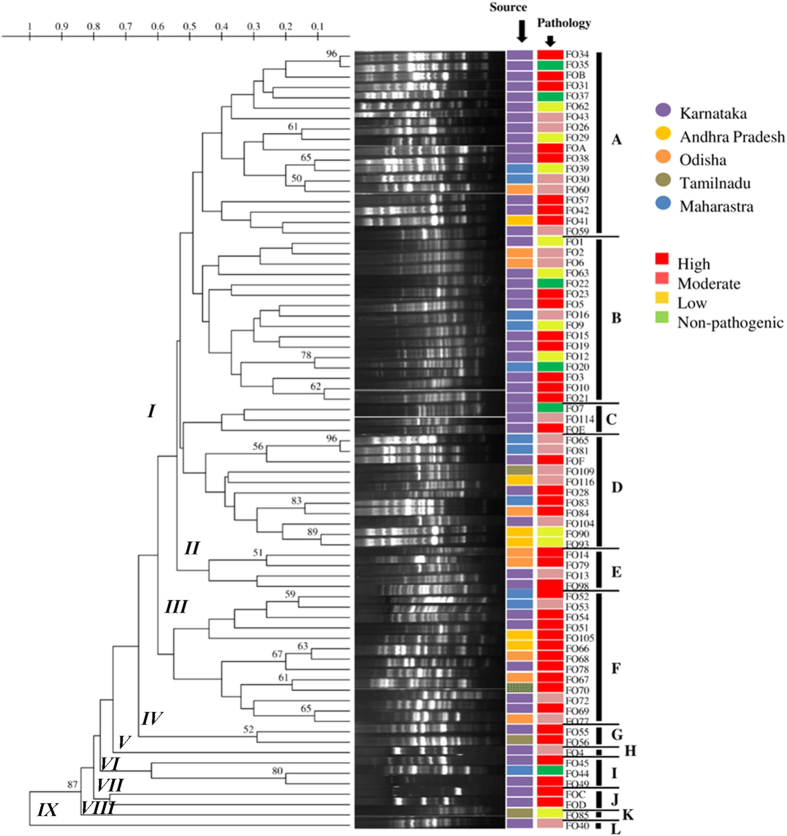
Phylogenetic analysis *F. oxysporum* f. sp. *lycopersici* isolates by ISSR analysis with Primer (GA)_9_C.

The genetic diversity of *F. oxysporum* strains was assessed by microsatellite based ISSR fingerprinting technique. A total of 10 ISSR primers were analysed for their capability to produce polymorphic amplicons on a subset of 25 strains including representatives from all the states in the study: Karnataka (39), Andhra Pradesh (6), Odisha (9), Tamilnadu (4) and Maharastra (11). Only 3 primers ((GA)_9_C, (GA)_9_T and (CAC)_3_GC) retained 100% typeability and resulted in robust and reproducible banding patterns for all the strains (Data not shown). Among these primers, two 3’ anchored microsatellites *viz*., (GA)_9_C and (GA)_9_T were utilized in the subsequent studies, the other primer was excluded as it generated smaller number of polymorphic bands allowing insufficient discrimination. The two primers (GA)_9_C and (GA)_9_T produced lucid band patterns of a range of approximately 03–26 bands and 02–22 bands, respectively per strain within a range of 100–3000 bp. Using both the primers, all the *F. oxysporum* strains used in the study were found typeable and UPGMA/Dice dendrograms drawn based on the band patterns showed high diversity among the strains. To determine the discriminatory power of the primers statistically, we estimated Simpson’s (SID) and Shannon’s (H) indices of diversity using Comparing Partitions online tool (Table 2). The calculated SID and H were found equivalent for both the primers at 95% Pearson correlation coefficient level, but on further analysis at lower correlation levels, (GA)_9_T primer was found more discriminative than (GA)_9_C primer. Based on the observation of the topology of the dendrograms, major branches of each dendrogram were assigned a roman numeral. The primer (GA)_9_T resulted in five such major groups (groups I to V) and (GA)_9_C resulted in nine groups (Groups I to IX) (Figs 2 and 3).

In the dendrogram generated by banding patterns of (GA)_9_T-ISSR-PCR, Clade I was the largest and was found to be differentiated into four distinct sub-clades; IA-ID. Majority of the *F. oxysporum* strains from Karnataka state were grouped in the sub-clades IA and IB (69%) and the rest were distributed across the dendrogram. Clade III had 6 strains and 5 of them were from Karnataka state. All the Odisha strains were clustered in sub-clades IB, ID, IIE and IIF. Strains from AP, TN and MH did not cluster as Karnataka strains and were distributed through the clades. One prominent detail in the dendrogram was one strain each from AP and TN had exactly similar banding pattern and clustered into single clade V and both these isolates were less pathogenic. Another notable difference in banding patterns obtained by (GA)_9_T-ISSR-PCR from clade V was the presence of an approximately 1300 bp dense band that was absent from the other isolate patterns. The most remarkable feature in the clustering observed in (GA)_9_T-ISSR-PCR was clade II included only pathogenic strains (Moderate and High) and had at least one representative strain from all the states in the study.

Alternatively, in the dendrogram generated based on banding patterns of (GA)_9_C-ISSR-PCR, clade I being the largest was differentiated again into four distinct sub-clades, IA-ID. Sub-clades IA, IB and IC contained majorly Karnataka strains and contrastingly only 3 out of 11 strains in ID were from Karnataka. No significant clustering was observed among the strains from different states. One noteworthy pattern observed in the clustering was that clades II, III and IV included only pathogenic strains from all the states and clade ID contained all pathogenic strains except two non-pathogenic strains from AP (FO90 and FO93).

**Figure 4:**
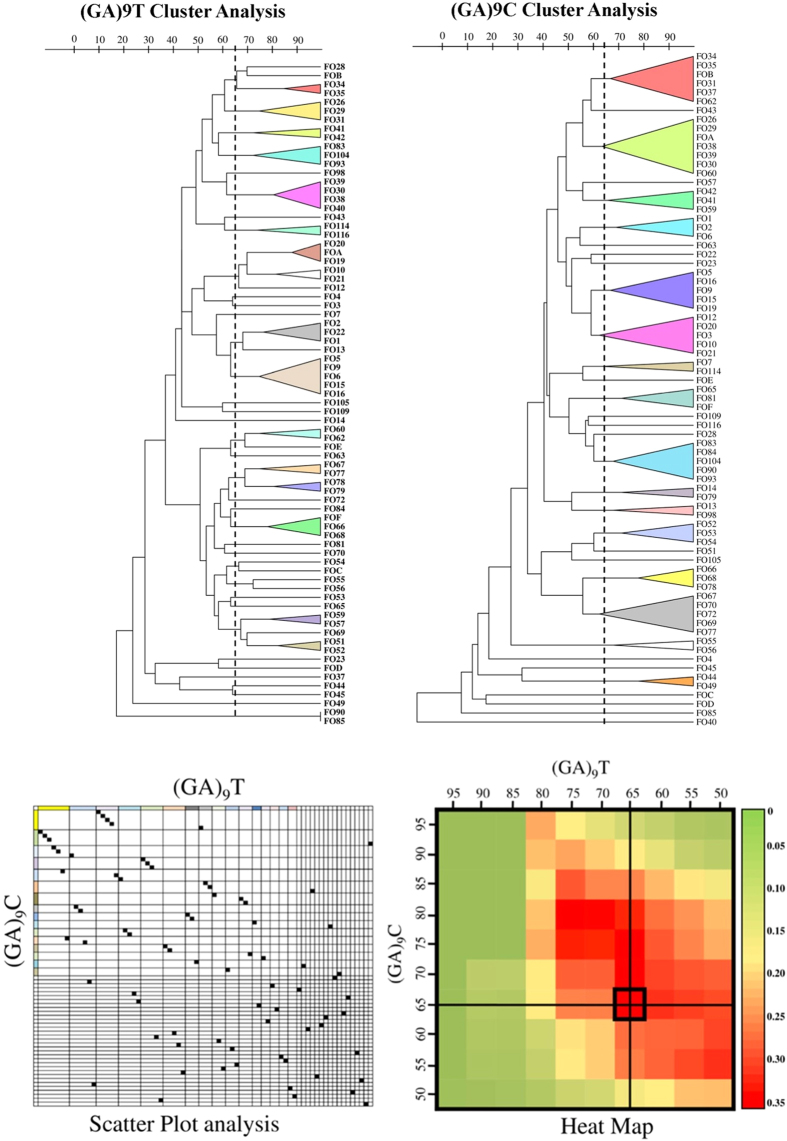
Comparative partition analysis- Diversity and partition congruence between the primers used in this study.

Our next objective was to identify the threshold that better defined the (GA)_9_T microsatellite partitions in comparison to those defined by (GA)_9_C. For each possible combination of partitions, the Adjusted Rand value was calculated by varying the threshold cutoffs for each UPGMA/Dice dendrograms of (GA)_9_C and (GA)_9_T from 50% to 95%. The maximum coefficient value was determined to be 0.278 at a threshold of 65% for both (GA)_9_C and (GA)_9_T dendrograms (Fig. 4). At this cutoff value, 33 clusters were defined by (GA)_9_C and 48 clusters by (GA)_9_T. SID values for the partitions found at this threshold level for (GA)_9_C and (GA)_9_T were 0.954 and 0.982, respectively. A scatter-plot was also constructed to visually represent the cluster congruence between (GA)_9_C and (GA)_9_T clusters and no significant congruence was observed except for four isolates FO5, FO9, FO15 and FO16. For comparing the congruence between type assignments of the (GA)_9_C and (GA)_9_T ISSR typing, Wallace coefficients were calculated at 65% cutoff value. Although a bidirectional correspondence, the correlation between the two typing techniques was weak. The Wallace values in the direction of (GA)_9_C and (GA)_9_T were 0.452 and 0.226, respectively. Considering this, the probability of two strains that shared a (GA)_9_T type sharing the same (GA)_9_C type was only 23% and in vice versa the value was 45%. On a whole, the weak Adjusted Rand and Wallace indices indicated a poor match between the partitions generated by (GA)_9_C and (GA)_9_T ISSR typing suggesting that both the microsatellites targeted different loci.

### ITS sequence analysis

The ITS region was successfully amplified from DNA from all *F. oxysporum* strains in the study by the fungal-specific universal primer pairs ITS1–ITS4. The lengths of sequences as determined by capillary electrophoresis ranged from 334 bp to 509 bp. The BLAST analysis of the ITS rDNA sequence data, supported the morphological identification, whereby the closest match (98–100% similarity) in the NCBI GenBank database was found to be with *F. oxysporum* and Fol. The ITS rDNA sequences of the 69 strains have been deposited in the NCBI GenBank database (GenBank Accession numbers KF914420- KF914488) (Table 1).

**Figure 5:**
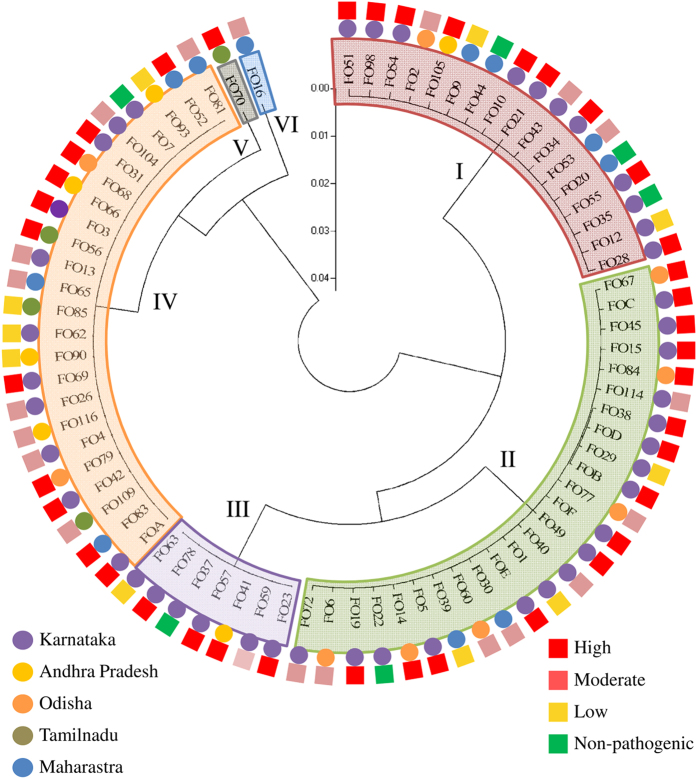
Phylogenetic analysis of *F. oxysporum* f. sp. *lycopersici* strains based on rDNA ITS sequences by Neighbor-Joining method. The tree includes reference strains of *F. oxysporum* f. sp. *lycopersici* and strains of other formae speciales.

A dendrogram was constructed based on the ITS sequences using ClustalW and Mega5 software by Neighbour-Joining method (Fig. 5). The phylogenetic tree proposed four major clades and two single membered clades with strains distributed across the dendrogram irrespective of their geographic and pathogenic status. Bootstrapping indicated that each branch corresponding to the particular clade was well supported (above 60%). Isolates from Karnataka, Maharashtra, Andhra Pradesh and Odisha were found distributed in the clades I, II and IV. Clade I was found to be heterogenous with isolates from many states and varied pathogenicity. Clade II contained isolates from Karnataka, Odisha and Maharastra states and most of these were pathogenic (high or moderate), except FO22, FO1, FO29 and FO39. Clade III had all the strains isolated from Karnataka except FO41; remarkably this strain was isolated from border region of Andhra Pradesh and Karnataka. Similar to clade I, clade IV was found to be heterogenous. The isolates from Tamilnadu were found distinctly in the clade IV and were absent in clades I, II and III. One pathogenic isolate each from TN and Maharashtra formed the single member clades V and VI, respectively. On a whole, clades I and III typically consisted of pathogenic isolates of Fol isolated in the present study. Interestingly, clade I, III, IV, V and VI uniquely consisted of *F. oxysporum* f. sp. *lycopersici* strains. Other formae speciales (reference isolates *F. oxysporum* f. sp. *ciceris*, *F. oxysporum* f. sp. *cepae*, *F. oxysporum* f. sp. *melonis* and *F. oxysporum* f. sp. *phaseoli*) were phylogenetically distinct from these clades and clustered only in the clade II along *F. oxysporum* f. sp. *lycopersici*. Fifty percent of the non-pathogenic strains were clustered in the clade I and one each were found in clades II, III and IV respectively.

To appreciate the polymorphism by ITS sequencing, we further studied the sequence analysis, upon which all the strains in the study displayed a general 80% sequence similarity which can be expected due to the conspecific nature of the strains. Further analysis revealed intraspecies variability and nucleotide insertions or deletions in the ITS1 region among the strains. Six variations were identified to be prominent in generation of the diversity observed in the dendrogram, as these variations were found to be clade specific (Supplementary File 1). In general, the variations in the ITS sequences include SNPs, *Taq*I restriction sites and oligos insertion/deletion termed A to F (Supplimentary File-2).

### Detection and Phylogeny of toxigenic *F. oxysporum* f. sp. *lycopersici*

A total of 31 highly pathogenic strains were screened to detect and identify fumonisin production. When the primer pair *Fum 1* was used, single band having amplicon size of ~790bp was observed for fumonisin producing strains of *F. oxysporum* (Fig. 1B). Among the 31 selected strains of pathogenic *F. oxysporum* f. sp. *lycopersici* tested, 11 isolates were found to be toxigenic harboring the *Fum1* gene. The present study reported clearly that some of the *F. oxysporum* strains infecting tomato have the potential to produce Fumonisin. In the cluster analysis based on ISSR PCR with the primer (GA)_9_C, the toxigenic strains were found distributed in the clades IA, IIE, IIIF and IVG. Interestingly, in the dendrogram generated with the primer (GA)_9_C, 60% of the toxigenic strains clustered in the clade IIF and the rest distributed in clades IA and IB. In the phylogenetic tree constructed with NJ based on ITS sequences, 60% of the Fumonisin positive strains clustered in the group IV and the rest were distributed equally in clades I and III.

## Discussion

Fusarium wilt of tomato has been reported in India since the late 1960’s but so far there is no comprehensive study on the genetic diversity of the pathogen which occurs virtually in all production areas of the country. In recent years, it has assumed serious proportions in the country due to the failure of recommended management practices made at present and farmers suffer from great economic losses. More recently, the interest in the pathogen has emerged due to increased problems experienced by farmers in tomato production fields. In addition to reporting the presence of Fol, the present study involved determining the genetic diversity within the pathogen population from major tomato growing regions of India.

Field survey revealed high incidence and the widespread prevalence of Fusarium wilt in the major tomato growing regions of India. The Fusarium wilt pathogen *Fusarium oxysporum* was commonly associated to diseased plants as well as soil samples in the affected fields. The high incidence of Fusarium wilt indicates that this disease is a recurrent problem and that all popular varieties appeared to be susceptible to the disease. Comparison of Fol strains isolated either from infected plants or rhizosphere soils, demonstrated a large diversity in terms of culture and morphology existing among strains of Fol. The strains varied widely in growth, colony characteristics, pigmentation and sporulation. The pathogenicity assay revealed that Fol strains could be categorized into four subgroups viz. non-pathogenic, low virulent, moderate virulent and high virulent strains. The pathogenic strains resulted in the development of the vascular wilt disease in the test tomato cultivars to various levels depending on their virulence. The non-pathogenic strains of *F. oxysporum* which are efficient colonizers of plant rhizosphere and roots, did not induce any symptoms. The results of the virulence study suggested the existence of a highly variable population of Fol. This study showed that 45% of the strains were highly virulent and 30% were moderately virulent which are distributed in almost all tomato growing areas of Southern India. This poses an important risk to the planting of local tomato varieties due to their susceptibility to the pathogen.

Accurate identification and genetic characterization of pathogens is necessary for appropriate management of plant diseases. The phylogenetic species concept has found relatively recent application in *Fusarium* systematics and can help to resolve taxonomic difficulties. The ISSR analysis is a robust, PCR-based technique that produces dominant molecular markers by DNA amplification of putative microsatellite regions^37^. ISSR markers detect a higher level of polymorphism than RAPD markers and have been used extensively in the analysis of fungal population. A high degree of genetic diversity among the Fol strains was observed based on ISSR analysis. Some of the strains formed region specific ISSR patterns and groups in the phylogenetic tree, whereas others from the same region showed distinct patterns and matched with the patterns of the strains of other regions. The ISSR markers clearly indicated the presence of highly variable populations within an area or state of India. However, some of the strains formed region specific ISSR patterns and groups in the phylogenetic tree. The genetic diversity observed among some strains obtained from populations in the same geographic locations were low. This limited variation might probably reflect the nature of the agricultural systems in these areas which greatly rely on the local varieties thus restricting the introduction of new genotypes of the pathogen.

The genetic diversity among the Fol strains of India revealed by ISSR analysis was in concordance with many earlier findings in *F. oxysporum* from different food crops^38^. Based on ISSR and other molecular markers, Dubey and Shio^39^ assessed the genetic diversity of chickpea wilt pathogen which revealed the presence of diverse populations and the occurrence of more than one race of the pathogen in the same locations. Their study indicated the existence of genetically diverse population of *F. oxysporum* f. sp. *ciceris* in different states of India. Baysal *et al.*^40^ used ISSR and RAPD markers to characterize *F. oxysporum* f. sp. *melongenae* isolates. The classification into groups based on ISSR and RAPD fingerprints showed genetic specificity and diversity among the isolates.

The ISSR primers yielded highly polymorphic bands and proved to be authentic and reliable markers for inferring the genetic relationships within the Fol strains. Phylogenetic analysis based on the fingerprints obtained through ISSR analysis indicated the presence of wide genetic diversity among Fol isolates of Southern India. It was evident from our results that ISSR is an efficient technique for the molecular typing of Fol strains and therefore this technique might replace other cumbersome fingerprinting methods for high throughput analysis.

In order to further understand the genetic relationship within the Fol strains, the ITS region was used as a genetic marker to investigate the phylogeny. Phylogenetic analysis based on the ITS sequences helped to reveal the evolutionary relationship within Fol and that with other formae speciales. In the present study, the phylogenetic tree based on the ITS sequence analysis revealed four major groups suggesting four major evolutionary lineages of Fol existing in tomato growing regions of India. Kawabe *et al.*^14^ studied a collection Fol isolates from Japan and other countries and reported three evolutionary lineages of Fol detected based on phylogenetic analysis of the ribosomal DNA intergenic spacer (rDNA IGS) region. Each lineage consisted of isolates mainly belonging to a single or closely related vegetative compatibility group (VCG) and a single mating type (MAT)^14^. Phylogenetic analyses of sequences of the elongation factor α (EF-1α) and *exo* polygalacturonase (*pgx*4) gene were conducted by Lievens *et al.*^29^ on a worldwide collection of Fol strains. Based on the reconstructed phylogenies, multiple evolutionary lineages were observed in Fol. In addition, mating type analysis showed a mixed distribution of the *MAT1-1* and *MAT1-2* alleles irrespective of the geographic origin of the isolates. Their study revealed that Fol was composed of at least three independent clonal lineages based on the EF-1α and *pgx*4 groupings.

Further, the clustering of *F. oxysporum* f. sp. *lycopersici* with non pathogenic isolates and with the members of other formae speciales such as *F. oxysporum* f. sp. *ciceris*, *F. oxysporum* f. sp. *cepae*, *F. oxysporum* f. sp. *melonis* and *F. oxysporum* f. sp. *phaseoli* was observed in our study. These results suggest that Fol is polyphyletic in origin. O’Donnell *et al.*^41^ showed that several formae speciales of *F. oxysporum*, including Fol were not monophyletic. In other words, isolates of a particular forma specialis might exhibit more close relatedness to some isolates from other formae speciales and non pathogenic strains of *F. oxysporum* than with the isolates of the same forma specialis^26^. Based on the present study Fol was found to be polyphyletic suggesting that the pathogenicity towards tomato had evolved several times independently^26^. These results are in accordance with previous observations of O’Donnell *et al.* and Cai *et al.*^26^,^41^.

There is a need for rapid, inexpensive and reliable diagnostic protocols for the detection of plant pathogen strains producing different mycotoxins. Although strains of *F. oxysporum*, do not typically produce fumonisins, there are several reports of fumonisin production by individual strains of this species^42^,^43^. PCR approaches based on primer sets targeted to toxin biosynthetic pathway genes have been reported for fumonisinfumonisin producing *Fusarium*^44^. In this study we attempted the detection of fumonisin producing Fol using the PCR based approach^45^. Of the 31 strains isolated from various regions of South India, the primers used detected 11 toxigenic strains of Fol. However, there is a need for further studies focusing on the natural occurrence of fumonisin in diseased tomato plants and fruits and its toxic effects upon consumption.

The ability to distinguish precisely between and within fungal species is crucial in developing a fingerprint database and in determining their emergence and evolution. Genetic differentiation detected within pathogen population can be assumed as environmental factors affecting pathogen properties^46^. The existence of mixed population for same forma specialis within a region could be due to the possible movement of infested soil on equipment, packing boxes or other items could have introduced the pathogen from one region to the other. *Fusarium oxysporum* f. sp. *lycopersici* may be disseminated in seed, transplants, or soil. The tomato crop is produced from seedlings obtained from seedling producers whose seeds are also provided from local and international seed companies^38^. Evidence from the present study, as well as from literature indicates that microevolutionary events (could be the changes in virulence) occur among strains of Fol belonging to genetically distinct populations. Such changes may occur due to the selection pressure imposed by resistance genes deployed on a large scale in commercial tomato varieties^24^. Several genes involved in the host defense systems, as well as virulence factors, have been shown to be under positive selection pressure and exhibit a faster accumulation of mutations compared to the genes regulating the basal physiology of the organism^47^. Adaptive evolutionary shifts of pathogen populations in response to variation in host genotype have been observed in many pathogen-host interactions, e.g., *F. oxysporum* f. sp. *lycopersici* on tomato^48^, *F. oxysporum* f. sp. *vasinfectum* on cotton^49^, *F. oxysporum* f. sp. dianthi on carnation^50^. Genetic diversity revealed within same races may be related with the difficulties in controlling the pathogen and the pathogen gaining resistance against the chemicals used in plant protection or may be associated with the exposure of the pathogens to abiotic stress^38^. Fourie *et al.*^51^ suggested that both coevolution with the host and horizontal gene transfer may play important roles in the evolutionary history of the pathogen. Although *F. oxysporum* is considered to be strictly mitotic, genetic exchange among and within individual lineages might occur more frequently than originally thought^52^. Genetic recombination could be due to parasexuality, a nonsexual mode of genetic exchange, or heterokaryosis, a process that is initiated by fusion of vegetative hyphae (anastomosis) between individuals with very similar genomes^51^.

## Conclusion

The present study repors the molecular characterization pathogenicity and toxigenicity of *F. oxysporum* f. sp. *lycopersici* strains originated from India and their relationship with other formae specilaes within the *F. oxysporum* complex. The study also indicated that analyzing the genetic variability among Fol strains would be of great importance in plant breeding for disease resistance and can be used by plant breeders. The molecular markers used in the study can be efficiently used for rapid and reliable identification and characterization of Fol population. Our investigation forms the basis for increasing the understanding of underlying genes related to host–pathogen interactions. Such information may help understand virulence dynamics of the pathogen, and facilitate development of efficient disease management strategies using gene-based molecular markers.

## Materials and Methods

### *Fusarium oxysporum* strains

Tomato growing areas in 5 states of South India (Karnataka, Andhra Pradesh, Tamilnadu, Maharashtra and Orissa) were surveyed and wilt infected plants and rhizosphere soil samples were collected from 72 different locations. For isolation of *Fusarium oxysporum* from wilted tomato plant samples, root and stem tissues were washed under running tap water. Plant pieces taken from the lower hypocotyls and upper taproot were surface sterilized in 1% NaOCl (Sodium hypochlorite) solution for 1 to 2 min, rinsed twice in sterile distilled water and dried between sterile filter papers. Pieces of surface disinfected tissues were placed on Potato Dextrose Agar (PDA) plates amended with 50 mg/L tetracycline to suppress the bacterial growth. The plates were incubated at room temperature for 7–10 days for the development of typical white mycelial growth of *Fusarium oxysporum* and subcultured onto PDA slants^53^. Isolation from rhizosphere soil samples was done by dilution plate technique. Soil dilutions were plated on PDA and incubated at 28 ± 2 °C for 5 days. Pure cultures were maintained on PDA slants at 4 °C and the lyophilized cultures were stored at −80 °C. The isolates were observed for cultural characters and morphology of the conidia. Colonies exhibiting the taxonomic features of *F. oxysporum* were identified according to Nelson *et al.*^54^. Morphological identification was based on characteristics of the macroconidia, phialides, microconidia, chlamydospores and colony growth traits.

### Pathogenicity testing of strains

To determine the formae specials, virulence analysis of the strains was carried out on a set of five tomato cultivars susceptible to Fusarium wilt. A total of 69 strains of *F. oxysporum* were tested for pathogenicity. Twenty day old healthy seedlings were inoculated by standard root dip method. Seedlings were uprooted carefully preserving the root integrity, shaken to remove the adhering particles and washed gently under tap water. The root apex (about 1 cm) was trimmed with a pair of sterile scissors and submerged for 30 minutes in the conidial suspension of each strain. Seedlings dipped in sterile water served as control. The inoculated seedlings and control were transplanted to minipots (15 cm diameter, surface sterilized with 0.1% mercuric chloride) containing sterilized soil and sand 1:1 ratio. Five seedlings per pot were transplanted. Plants were maintained in a greenhouse where day and night temperatures varied between 25–30 °C. Seedlings were watered daily and fertilized once with NPK (15:15:15). Symptoms started to be visible 15–20 days after artificial inoculation. Disease severity was assessed from 2 weeks of inoculation up to 45 days. The disease index used throughout the experiments ranged from 0 to 100% (0- healthy plant; 25- initial symptoms of leaf chlorosis; 50- severe leaf chlorosis and initial symptoms of wilting; 75-severe wilting symptoms and leaf chlorosis; 100-plant totally wilted, leaves completely necrotic). The discoloration of the vascular tissue was confirmed by slitting the stem^24^,^39^. Data were statistically analyzed by using analysis of variance (ANOVA) and Duncan’s test (*P* < 0.05)^55^.

### DNA Extraction

Fungal strains were grown for 5 days in Potato Dextrose Broth (PDB) at 28 °C under static conditions. Mycelia were harvested and lyophilized. Later 100 mg of the finely powdered mycelium was used for DNA extraction using Hi PurA^TM^ Fungal DNA Mini Kit (HiMedia, India), according to the manufacturer’s instructions. The concentration and purity of extracted DNA was determined using NanoDrop-ND-1000 spectrophotometer (Nanodrop Technologies, Wilmington, USA) and the DNA samples were stored at −80 °C for further use.

### Species specific PCR assay

Species specific PCR assay was carried out by using the species specific primers as described by Prashant *et al.*^56^ (Table 3). The PCR amplification was performed in 20 μL reaction which contained 2 μL of 10× PCR buffer, 2 mM MgCl_2_, 100 μM dNTPs, 1 unit of *Taq* polymerase (MBI Fermentas, India) and 20 ηg of template DNA in a Mastercycler thermal cycler (Eppendorf, Hamburg, Germany). The PCR programme consisted of initial denaturation at 94 °C for 4 min followed by 30 cycles of denaturation at 94 °C for 1 min, annealing at 58 °C for 1 min and extension at 72 °C for 2 min, and final extension at 72 °C for 10 min. PCR amplicons were resolved on 1% agarose gel and visualized in a UV transilluminator. A 1 kb ladder was used as a marker. Gels were documented using Gbox (GE health care, Mumbai).

### Detection of toxigenic *F. oxysporum* f. sp. *lycopersici* strains

Primers were used for specific detection of potentially toxigenic *F. oxysporum* f. sp. *lycopersici* strains by targeting the metabolic pathway genes specific to *fum1* for detection of fumonisin producing strains as reported earlier by Ramana *et al*.(2011) 44 (Table 3). PCR was carried out in an Eppendorf master cycler gradient (Hamburg, Germany, MastercylcerR pro 384) with a reaction volume of 30 μL. The amplification mixture consisted of template DNA (1.0 μL), 2 mM MgCl_2_, 1× PCR buffer, 200 μM dNTP mix, 1.0 unit *Taq* polymerase and primer pairs specific to the targeted gene (*fum1*) were added at a concentration of 100 nM to the each individual reaction. The PCR was carried out with an initial denaturation at 94 °C for 4 min, followed by 30 cycles of 94 °C for 1 min, 58 °C for 1 min and 72 °C for 1.5 min with a final extension of 72 °C for 8 min. After successful amplification, PCR products were loaded onto 1% agarose gel containing ethidium bromide and visualized under UV.

### Genetic diversity among *F. oxysporum f.* sp. *lycopersici* strains

#### Inter Simple Sequence Repeat (ISSR) PCR Analysis

The 69 *F. oxysporum* f. sp. *lycopersici* strains subjected to pathogenicity testing were subjected to ISSR analysis. A total of 10 ISSR primers were synthesized from Eurofins, Bangalore, India (Table 4) and used for the amplification of microsatellite loci. To select primers which produce polymorphic and reproducible bands for the characterization of the strains, the 10 ISSR primers with di-or tri nucleotide repeats were screened using DNA samples from representative strains. Among the primers tested, 2 primers (GA)_9_T and (GA)_9_C were selected based upon the production of distinct reproducible polymorphic banding patterns. PCR reactions were performed using an Eppendorf master cycler gradient (Hamburg, Germany, MastercylcerR pro 384). PCR was carried out in a 20 μL reaction volume with 2.0 μL of 10× Taq buffer, 0.2 mM dNTPs (2.5 mM each), 2.0 mM MgCl_2_, 10 pM of each primer, 1.0 U of *Taq* DNA polymerase and 50 ng template DNA. For each primer-strain combination, amplifications were repeated at least thrice to assure reproducibility. Negative controls using sterile water were included in each experiment. The PCR cycling conditions were carried out with an initial denaturation at 94 °C for 3 min, followed by 35 cycles of 94 °C for 1 min, 45 °C for 1 min and 72 °C for 2 min with a final extension of 72 °C for 10 min. Amplification products were electrophoresed in 1.5% Agarose gel in 0.5× Tris base EDTA buffer at 60 V cm^−1^.

### Data Analysis

For ISSR analysis, the presence or absence of an allele at a particular locus was scored as 1 and 0 respectively and the pairwise distance among the strains was calculated from the binary matrix using the Jaccard’s coefficient. The resulting distance matrices were used to cluster the strains by the UPGMA (Unweighted Pair Group method using arithmetic means) method.

### ITS amplification

The ITS region of the rDNA was amplified using primers ITS1 and ITS4 (White *et al.* 1990) (Table 3). Amplification reactions were performed in a total volume of 30 μL containing 1× PCR buffer, 200 μM each dNTPs, 1.4 mM MgCl_2_, 1 μL of each primer (100 nmol), 1.0 U *Taq* DNA polymerase, and 20 ng of genomic DNA. PCR amplification conditions included an initial denaturation step at 94 °C for 4 min, cycling conditions were 94 °C for 45 s, 56 °C for 45 s, 72 °C for 1 min (30 cycles), followed by a final extension at 72 °C for 5 min. Each set of the experiment included negative controls (without template DNA) to test the presence of contaminating DNA in reagents. The amplicons were checked in 1% agarose gels run parallel to standard DNA molecular weight marker.

### ITS Sequence analysis

The amplified ITS rDNA region of selected strains were purified using QIAquick PCR purification kit (QIAGEN, Germany), according to the manufacturer’s instructions. The amplified DNA fragments were then labelled using a Big dye terminator v3.1 cycle sequencing kit and sequenced on a 3730 × l DNA analyzer. The sequences were compared to those in GenBank (http://www.ncbi.nlm.nih.gov/) using Mega BLAST. The BLAST analysis was performed with full length ITS sequences as queries to reveal relationships to published sequences. Highest homology and total score were noted for further analysis. The sequences obtained in the present study were submitted to GenBank. The ITS sequences of *F. oxysporum* f. sp. *lycopersici* and *F. oxysporum* strains from other formae speciales were downloaded from the NCBI GenBank database and were used in the phylogenetic analyses as reference sequences. All the DNA sequences were aligned with the program Clustal W included in BioEdit sequence alignment editor^57^,^58^. The resulting multiple-alignment file was used for phylogenetic analyses which were performed using Mega 5.0 with Neighbor-Joining method^59^.

### ITS-RFLP

The ITS PCR products from each of the Fol strains (10 μL) were digested individually with *Eco*RI restriction enzyme. The reaction mixtures were incubated overnight with the restriction enzyme at 37 °C overnight. The digested products were separated by electrophoresis on 2.0% agarose gel in 0.5 × TBE. The size of the separated fragments was determined by running in parallel a 100 bp DNA ladder (Sigma). Gels were stained with ethidium bromide (0.5 μg ml^−1^) to visualize the DNA under ultraviolet light and gel images were obtained with a Gel Doc System.

## Additional Information

**How to cite this article**: Nirmaladevi, D. *et al.* Molecular phylogeny, pathogenicity and toxigenicity of *Fusarium oxysporum* f. sp. *lycopersici*. *Sci. Rep.* **6**, 21367; doi: 10.1038/srep21367 (2016).

## Supplementary Material

Supplementary Information

## Figures and Tables

**Table 1:** Strains of *Fusarium oxysporum* f. sp. *lycopersici* and non pathogenic strains used for virulence and genetic diversity analysis through the application of ISSR and ITS Sequence analysis.

Strain No.	Source (Host/Habitat)	Geographic origin	Disease severity observed in tomato cultivars (Disease index 0-100%)						Virulence grade	GenBankAccession No
			Arka Abha	Arka Alok	Arka Meghali	Arka Saurabh	ArkaVikas	Mean		
FO38	Stem	Karnataka	73.33^abc^	80.00^abc^	80.00^abc^	80.00^ab^	80.00^a^	78.67	High	KF914420
FO84	Stem	Odisha	53.33^cdef^	60.00^cdef^	60.00^cdef^	60.00^bcde^	46.67^cde^	56	High	KF914421
FO81	Stem	Maharashtra	13.33^hi^	40.00^fgh^	40.00^fg^	40.00^ef^	13.33^fg^	29.33	Moderate	KF914422
FO4	Stem	Karnataka	40.00^efg^	53.33^defg^	53.33^def^	40.00^ef^	40.00^de^	45.33	Moderate	KF914423
FO3	Stem	Karnataka	66.67^abcd^	73.33^abcd^	73.33^abcd^	66.67^abcd^	66.67^abc^	69.33	High	KF914424
FO2	Rhizosphere Soil	Odisha	13.33^hi^	26.67^hi^	40.00^fg^	26.67^fg^	26.67^ef^	26.67	Moderate	KF914425
FO66	Rhizosphere Soil	Andhra Pradesh	53.33^cdef^	60.00^cdef^	46.67^efg^	40.00^ef^	53.33^bcd^	50.67	High	KF914426
FO93	Rhizosphere Soil	Andhra Pradesh	13.33^hi^	0^j^	26.67^gh^	0^h^	40.00^de^	16	Low	KF914427
FO56	Rhizosphere Soil	Tamilnadu	66.67^abcd^	80.00^abc^	66.67^bcde^	46.67^def^	60.00^abcd^	64	High	KF914428
FO52	Stem	Maharashtra	13.33^hi^	0^j^	0^i^	40.00^ef^	0^g^	10.67	Low	KF914429
FO55	Rhizosphere Soil	Karnataka	0^i^	13.33^ij^	13.33^hi^	13.33^gh^	0^g^	8	Low	KF914430
FO44	Rhizosphere Soil	Maharashtra	0^i^	0^j^	0^i^	0^h^	0^g^	0	Non pathogenic	KF914431
FO85	Rhizosphere Soil	Tamilnadu	13.33^hi^	13.33^ij^	13.33^hi^	13.33^gh^	13.33^fg^	13.33	Low	KF914432
FO14	Stem	Odisha	80.00^ab^	80.00^abc^	80.00^abc^	73.33^abc^	80.00^a^	78.67	High	KF914433
FO26	Rhizosphere Soil	Karnataka	33.33^fgh^	46.67^efgh^	13.33^hi^	40.00^ef^	46.67^cde^	36	Moderate	KF914434
FO77	Rhizosphere Soil	Odisha	26.67^gh^	53.33^defg^	53.33^def^	40.00^ef^	13.33^fg^	37.33	Moderate	KF914435
FO13	Rhizosphere Soil	Karnataka	40.00^efg^	40.00^fgh^	40.00^fg^	40.00^ef^	40.00^de^	40.00	Moderate	KF914436
FO34	Stem	Karnataka	53.33^cdef^	60.00^cdef^	53.33^def^	60.00^bcde^	46.67^cde^	54.67	High	KF914437
FO43	Stem	Karnataka	60.00^bcde^	46.67^efgh^	46.67^efg^	46.67^def^	40.00^de^	48	Moderate	KF914438
FO28	Rhizosphere Soil	Karnataka	66.67^abcd^	80.00^abc^	73.33^abcd^	66.67^abcd^	73.33^ab^	72	High	KF914439
FO35	Stem	Karnataka	0^i^	0^j^	0^i^	0	0^g^	0	Non pathogenic	KF914440
FO37	Stem	Karnataka	0^i^	0^j^	0^i^	0^h^	0^g^	0	Non pathogenic	KF914441
FO60	Rhizosphere Soil	Odisha	40.00^efg^	40.00^fgh^	13.33^hi^	40.00^ef^	26.67^ef^	32	Moderate	KF914442
FO63	Rhizosphere Soil	Karnataka	60.00^bcde^	53.33^defg^	46.67^efg^	60.00^bcde^	60.00^abcd^	56	High	KF914443
FO62	Stem	Karnataka	0^i^	0^j^	0^i^	0^h^	13.33^fg^	2.67	Low	KF914444
FO65	Stem	Maharashtra	13.33^hi^	40.00^fgh^	40.00^fg^	40.00^ef^	13.33^fg^	29.33	Moderate	KF914445
FO68	Rhizosphere Soil	Odisha	73.33^abc^	80.00^abc^	66.67^bcde^	73.33^abc^	73.33^ab^	73.33	High	KF914446
FO1	Stem	Karnataka	13.33^hi^	26.67^hi^	13.33^hi^	0^h^	0^g^	10.66	Low	KF914447
FO9	Stem	Maharashtra	0^i^	0^j^	26.67^gh^	13.33^gh^	0^g^	8	Low	KF914448
FO22	Rhizosphere Soil	Karnataka	0^i^	0^j^	0^i^	0^h^	0^g^	0	Non pathogenic	KF914449
FO23	Rhizosphere Soil	Karnataka	73.33^abc^	80.00^abc^	66.67^bcde^	60.00^bcde^	80.00^a^	72	High	KF914450
FO41	Rhizosphere Soil	Andhra Pradesh	80.00^ab^	86.67^ab^	86.67^ab^	86.67^a^	80.00^a^	84	High	KF914451
FO30	Rhizosphere Soil	Maharashtra	40.00^efg^	46.67^efgh^	46.67^efg^	26.67^fg^	40.00^de^	40.00	Moderate	KF914452
FO57	Rhizosphere Soil	Karnataka	53.33^cdef^	66.67^bcde^	60.00^cdef^	73.33^abc^	60.00^abcd^	62.67	High	KF914453
FO6	Rhizosphere Soil	Odisha	13.33^hi^	40.00^fgh^	40.00^fg^	13.33^gh^	40.00^de^	29.33	Moderate	KF914454
FO21	Stem	Karnataka	73.33^abc^	80.00^abc^	80.00^abc^	66.67^abcd^	73.33^ab^	74.67	High	KF914455
FO19	Root	Karnataka	73.33^abc^	80.00^abc^	80.00^abc^	66.67^abcd^	80.00^a^	76	High	KF914456
FO51	Stem	Karnataka	13.33^hi^	13.33^ij^	0^i^	13.33^gh^	0^g^	8	Low	KF914457
FO16	Rhizosphere Soil	Maharashtra	46.67^defg^	46.67^efgh^	13.33^hi^	40.00^ef^	46.67^cde^	38.67	Moderate	KF914458
FO78	Stem	Karnataka	80.00^ab^	93.33^a^	93.33^a^	80.00^ab^	80.00^a^	85.33	High	KF914459
FO59	Rhizosphere Soil	Karnataka	33.33^fgh^	40.00^fgh^	40.00^fg^	0^h^	13.33^fg^	25.33	Moderate	KF914460
FO49	Stem	Karnataka	80.00^ab^	80.00^abc^	80.00^abc^	80.00^ab^	80.00^a^	80.00	High	KF914461
FO54	Stem	Karnataka	60.00^bcde^	66.67^bcde^	66.67^bcde^	66.67^abcd^	53.33^bcd^	62.67	High	KF914462
FO45	Rhizosphere Soil	Karnataka	66.67^abcd^	53.33^defg^	60.00^cdef^	53.33^cde^	53.33^bcd^	57.33	High	KF914463
FO79	Stem	Odisha	80.00^ab^	73.33^abcd^	73.33^abcd^	80.00^ab^	80.00^a^	77.33	High	KF914464
FO69	Root	Karnataka	73.33^abc^	66.67^bcde^	73.33^abcd^	80.00^ab^	60.00^abcd^	70.67	High	KF914465
FO70	Rhizosphere Soil	Tamilnadu	60.00^bcde^	60.00^cdef^	53.33^def^	60.00^bcde^	60.00^abcd^	58.67	High	KF914466
FO67	Stem	Odisha	80.00^ab^	73.33^abcd^	80.00^abc^	80.00^ab^	80.00^a^	78.67	High	KF914467
FO29	Stem	Karnataka	0	13.33^ij^	13.33^hi^	0^h^	0^g^	5.33	Low	KF914468
FO72	Stem	Karnataka	46.67^defg^	40.00^fgh^	26.67^gh^	26.67^fg^	53.33^bcd^	38.67	Moderate	KF914469
FO7	Rhizosphere Soil	Karnataka	0^i^	0^j^	0^i^	0^h^	0^g^	0	Non pathogenic	KF914470
FO10	Stem	Karnataka	60.00^bcde^	53.33^defg^	53.33^def^	60.00^bcde^	66.67^abc^	58.67	High	KF914471
FO39	Stem	Maharashtra	13.33^hi^	0^j^	13.33^hi^	0^h^	26.67^ef^	10.67	Low	KF914472
FO31	Rhizosphere Soil	Karnataka	53.33^cdef^	66.67^bcde^	66.67^bcde^	53.33^cde^	73.33^ab^	62.67	High	KF914473
FO12	Stem	Karnataka	13.33^hi^	0^j^	0^i^	0^h^	0^g^	2.67	Low	KF914474
FO5	Stem	Karnataka	53.33^cdef^	60.00^cdef^	60.00^cdef^	60.00^bcde^	53.33^bcd^	57.33	High	KF914475
FO15	Stem	Karnataka	80.00^ab^	86.67^ab^	86.67^ab^	86.67^a^	80.00^a^	84	High	KF914476
FO105	Rhizosphere Soil	Andhra Pradesh	73.33^abc^	66.67^bcde^	60.00^cdef^	66.67^abcd^	66.67^abc^	66.67	High	KF914477
FO53	Stem	Maharashtra	40.00^efg^	40.00^fgh^	26.67^gh^	26.67^fg^	40.00^de^	34.67	Moderate	KF914478
FO42	Stem	Karnataka	53.33^cdef^	66.67^bcde^	53.33^def^	60.00^bcde^	53.33^bcd^	57.33	High	KF914479
FO20	Stem	Maharashtra	0^i^	0^j^	0^i^	0^h^	0^g^	0	Non pathogenic	KF914480
FO40	Stem	Karnataka	46.67^defg^	40.00^fgh^	40.00^fg^	53.33^cde^	60.00^abcd^	48	Moderate	KF914481
FO114	Rhizosphere Soil	Karnataka	46.67^defg^	40.00^fgh^	60.00^cdef^	40.00^ef^	40.00^de^	45.33	Moderate	KF914482
FO109	Rhizosphere Soil	Tamilnadu	46.67^defg^	40.00^fgh^	53.33^def^	46.67^def^	60.00^abcd^	49.33	Moderate	KF914483
FO98	Stem	Karnataka	60.00^bcde^	53.33^defg^	60.00^cdef^	53.33^cde^	60.00^abcd^	57.33	High	KF914484
FO90	Rhizosphere Soil	Andhra Pradesh	40.00^efg^	0^j^	13.33^hi^	0^h^	40.00^de^	18.67	Low	KF914485
FO83	Rhizosphere Soil	Maharashtra	53.33^cdef^	60.00^cdef^	53.33^def^	53.33^cde^	60.00^abcd^	56	High	KF914486
FO116	Rhizosphere Soil	Andhra Pradesh	46.67^defg^	33.33^ghi^	60.00^cdef^	46.67^def^	40.00^de^	45.33	Moderate	KF914487
FO104	Rhizosphere Soil	Karnataka	40.00^efg^	40.00^fgh^	40.00^fg^	26.67^fg^	40.00^de^	37.33	Moderate	KF914488

Disease severity observed in test susceptible tomato cultivars and virulence grade of *Fusarium oxysporum* strains.

^*^Disease index for the strains listed, assessed on a 0 to 100% scale. Analysis of variance (ANOVA) was performed and within each column, mean values followed by a common letter do not differ statistically according to DMRT (P < 0.05).

0: healthy plants; 12.5: plants growing regularly with slight vascular discoloration; 25: slight leaf chlorosis and reduced growth, vascular discoloration; 50: chlorosis, 50% growth reduction in respect to the healthy control, vascular discoloration, initial symptoms of wilting; 75: extended vascular discoloration, strong leaf chlorosis, severe growth reduction and wilting symptoms; 100: plants totally wilted and dead.

**Table 2:** Observed subtypes, Simpson’s index of diversity (SID) with jackknife pseudo-values at confidence interval of 95% and entropy (H) at different correlation levels for ISSR typing with two primers used in the study.

ISSR Primer	Correlation level(%)	# Partitions	SID	Jackknife pseudo-values CI (95%)	H
(GA)_9_C	95	72	0.999	(0.998–1.000)	6.155
	90	71	0.999	(0.997–1.000)	6.122
	80	59	0.993	(0.988–0.998)	5.765
	70	42	0.978	(0.968–0.988)	5.114
	60	24	0.927	(0.897–0.956)	4.025
(GA)_9_T	95	73	0.999	(0.998–1.000)	6.175
	90	70	0.998	(0.996–1.000)	6.095
	80	66	0.996	(0.991–1.000)	5.959
	70	53	0.989	(0.982–0.995)	5.559
	60	41	0.978	(0.969–0.987)	5.096

**Table 3:** List of primers used for identification of *F.oxysporum* f. sp. *lycopersici*, fumonisins gene and ITS analysis.

Primer	Primer Sequence	Amplicon size	Reference
FO1	5′-ACATACCACTTGTTGCCTCG-3′	340 bp	Prashant *et al.*2003
FO2	5′-CGCCAATCAATTTGAGGAACG-3′		
Fum1F	5′-ATTATGGGCATCTTACCTGGAT-3′	798 bp	Ramana *et al.*2011
Fum1R	5′-ACGCAAGCTCCTGTGACAGA-3′		
ITS1	5′-TCCGTAGGTGAACCTGCGG-3′	600 bp	White *et al.* 1990
ITS4	5′-TCCTCCGCTTATTGATATGC-3′		

**Table 4:** ISSR primers used in the present study to check the genetic diversity among strains of *F. oxysporum* f. sp. *lycopersici*.

Sl No.	PrimerName	Primer Sequence	
1.	ISSR-1	CACACACACACAGT	(CA)_6_GT
2.	ISSR-2	CTCTCTCTCTCTCTCTTC	(CT)_8_TC
3.	ISSR-3	CACCACCACGC	(CAC)_3_GC
4.	ISSR-4	CACACACACACACACAA	(CA)_8_A
5.	ISSR-5	CACACACACACAAG	(CA)_6_AG
6.	ISSR-6	GAGAGAGAGAGAGG	(GA)_6_GG
7.	ISSR-7	CTCTCTCTCTCTCTCTGC	(CT)_8_GC
8.	ISSR-8	GTGGTGGTGGTGGTG	(GTG)_5_
9.	ISSR-9	**GAGAGAGAGAGAGAGAGAC**	**(GA)**_**9**_**C**
10.	ISSR-10	**GAGAGAGAGAGAGAGAGAT**	**(GA)**_**9**_**T**
