# Supplementary material for: Molecular phylogeny, pathogenicity and toxigenicity of Fusarium oxysporum f. sp. lycopersici
Source: Sci Rep. 2016 Feb 17;6:21367. doi: 10.1038/srep21367 (PMC4756691; doi:10.1038/srep21367)

***Supplementary***

**Molecular phylogeny, pathogenicity and toxigenicity of *Fusarium oxysporum* f. sp. *lycopersici***

Nirmaladevi D^#1^, Venkataramana M^#2^, Rakesh K. Srivastava^3^ Uppalapati SR^4^, Vijai Kumar Gupta^5^, Yli-Mattila T^6^, Clement Tsui KM^7^, Srinivas C^1^, Niranjana SR^8^ and Chandra Nayaka S^8^*

**Figure. 1:** CLUSTALW alignment and SNPs identification


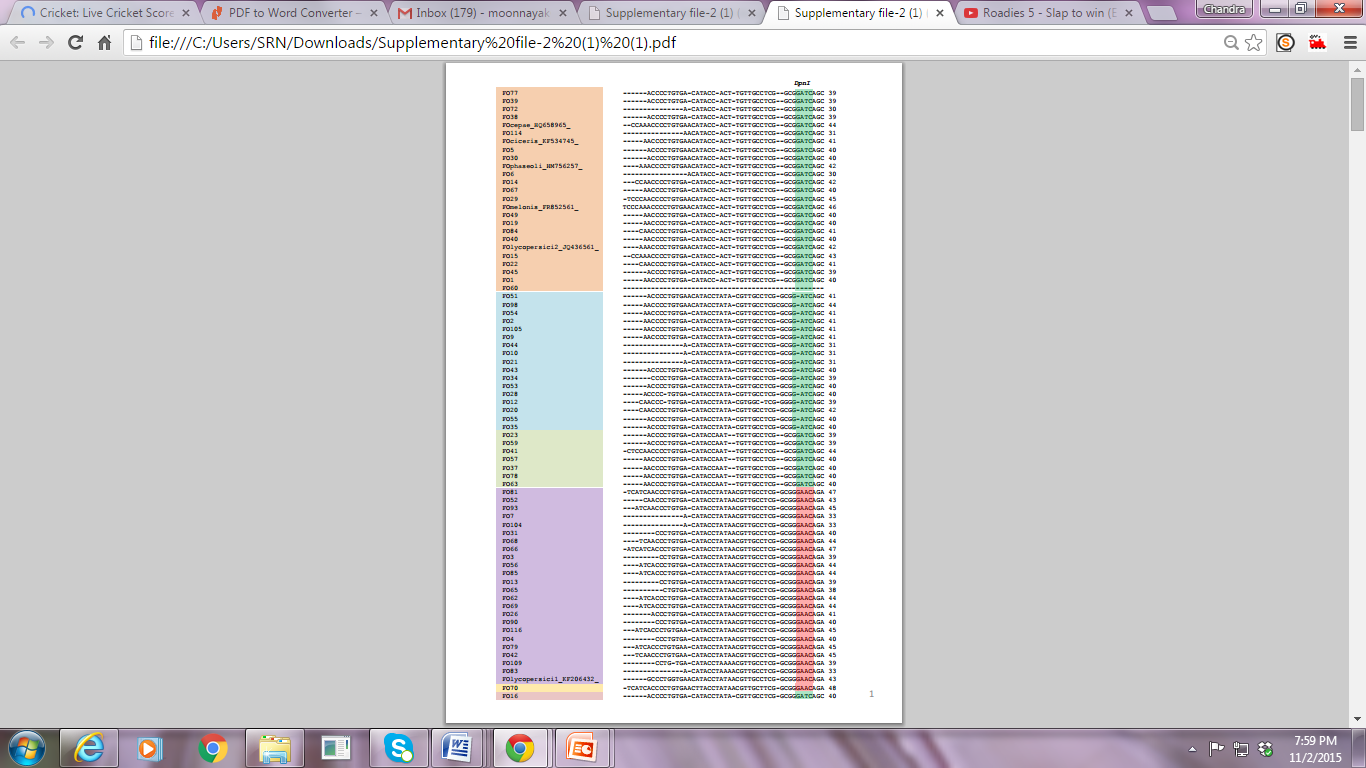


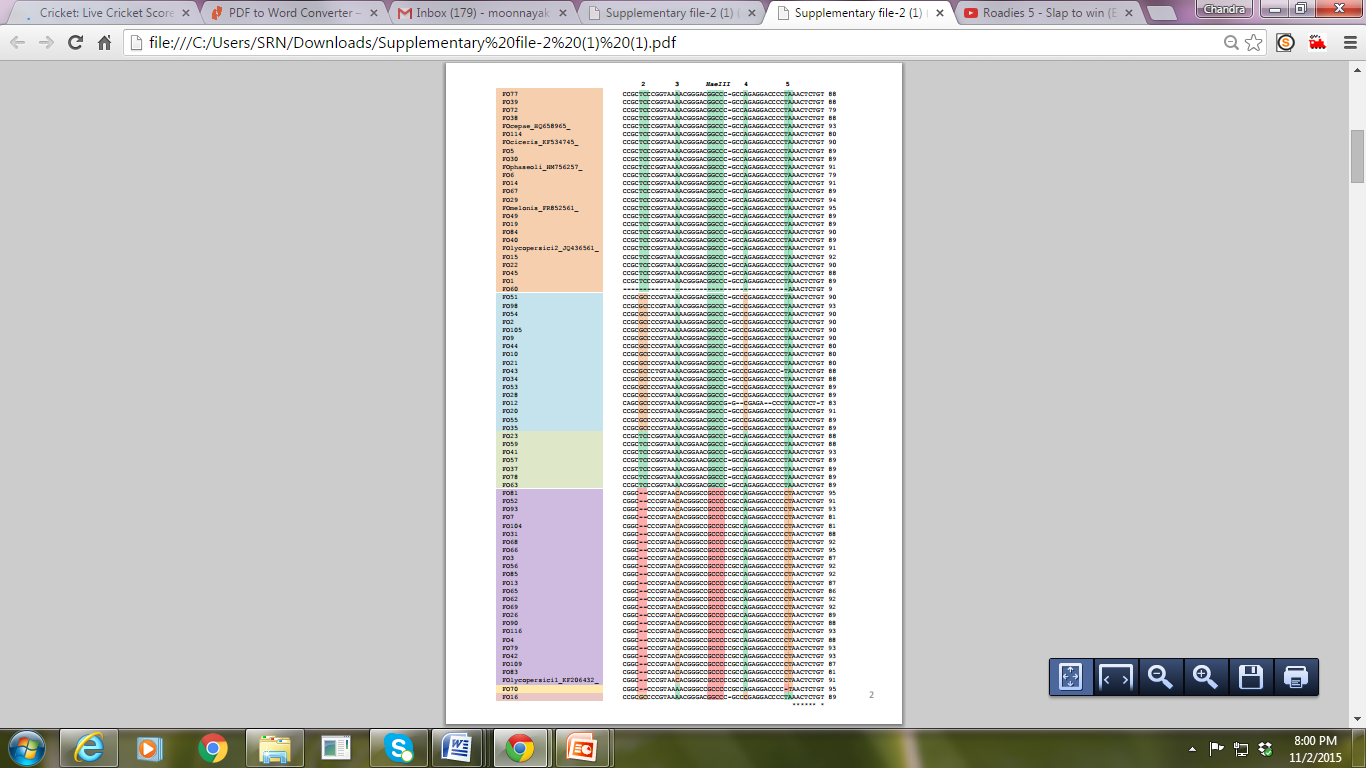


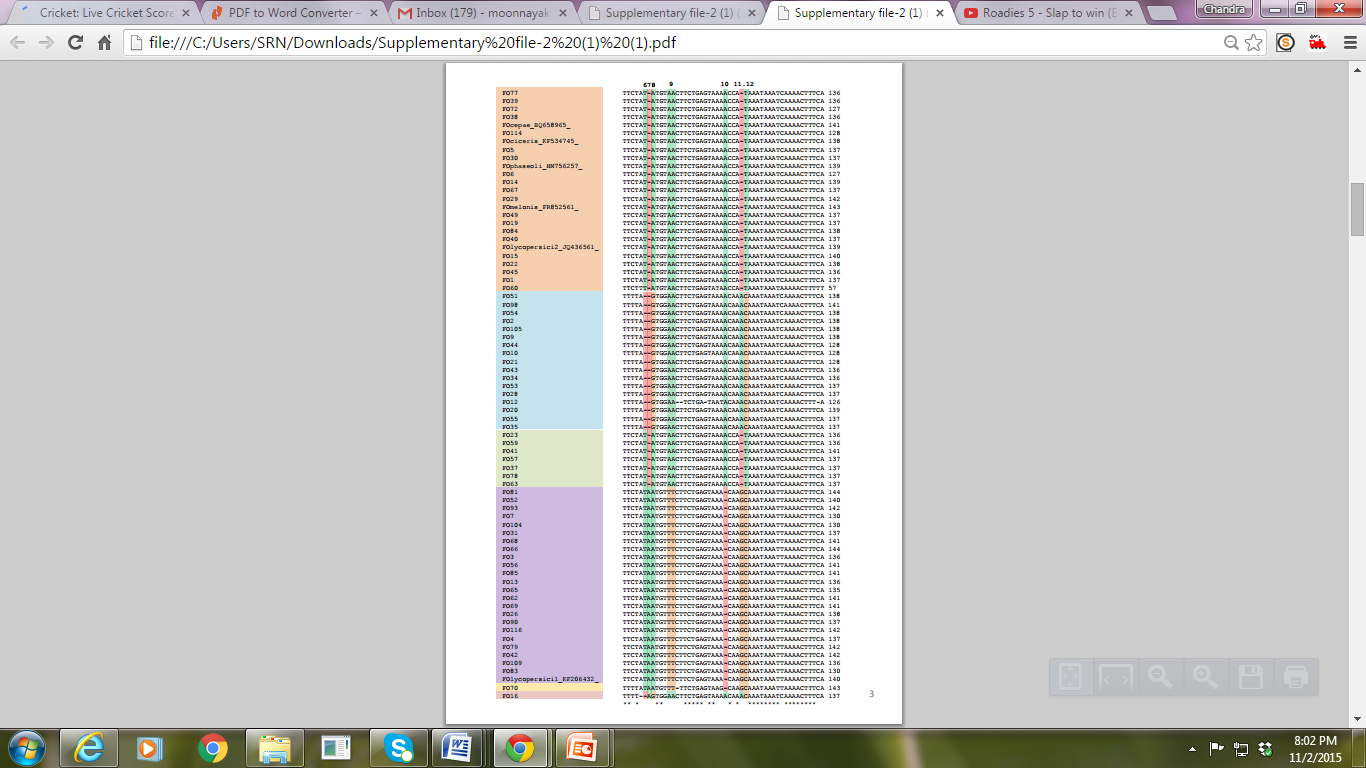


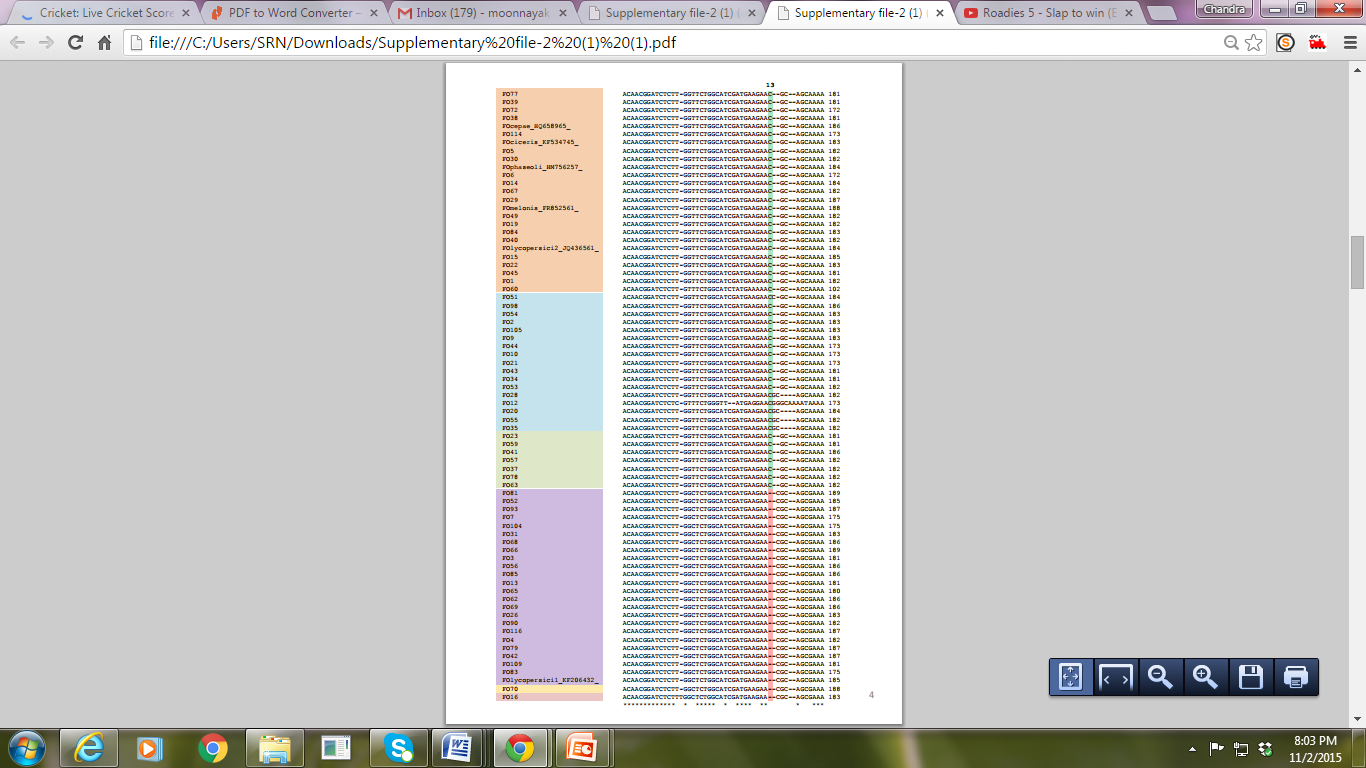


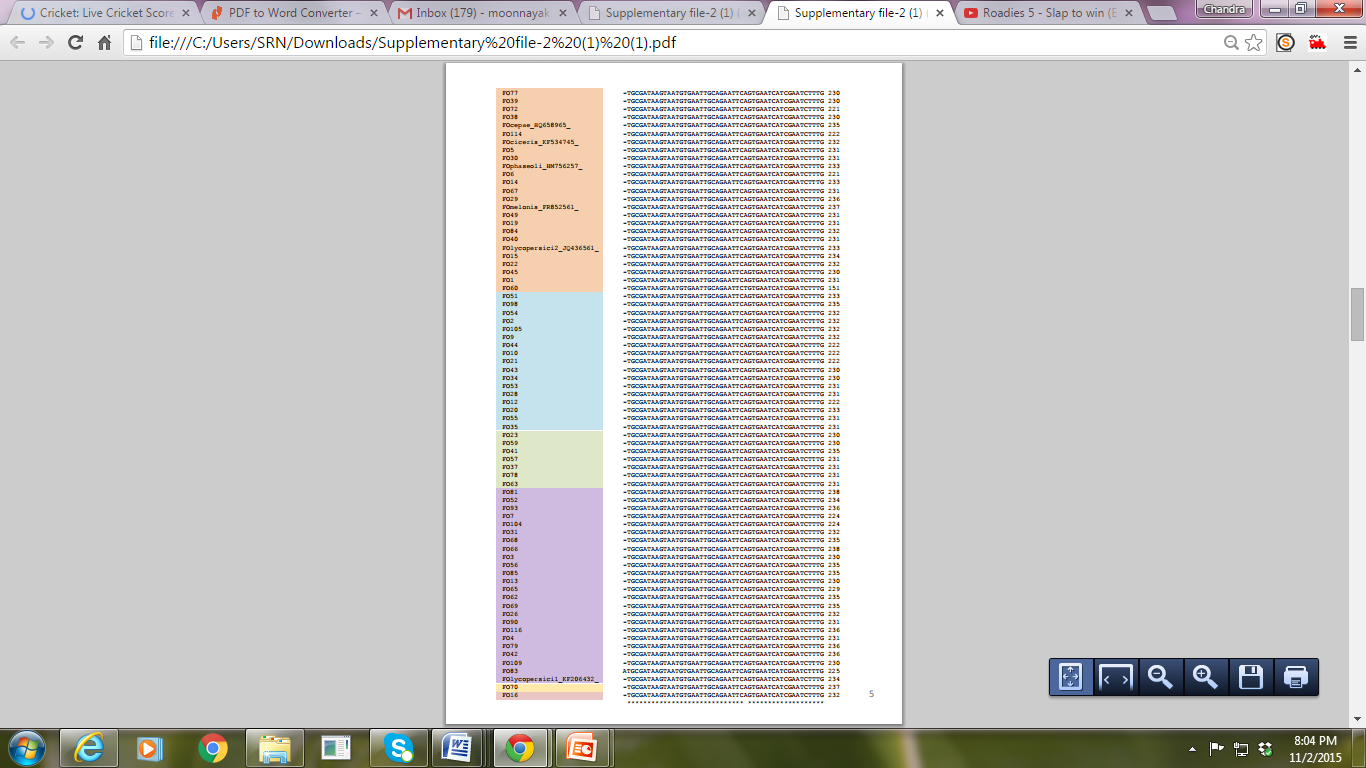


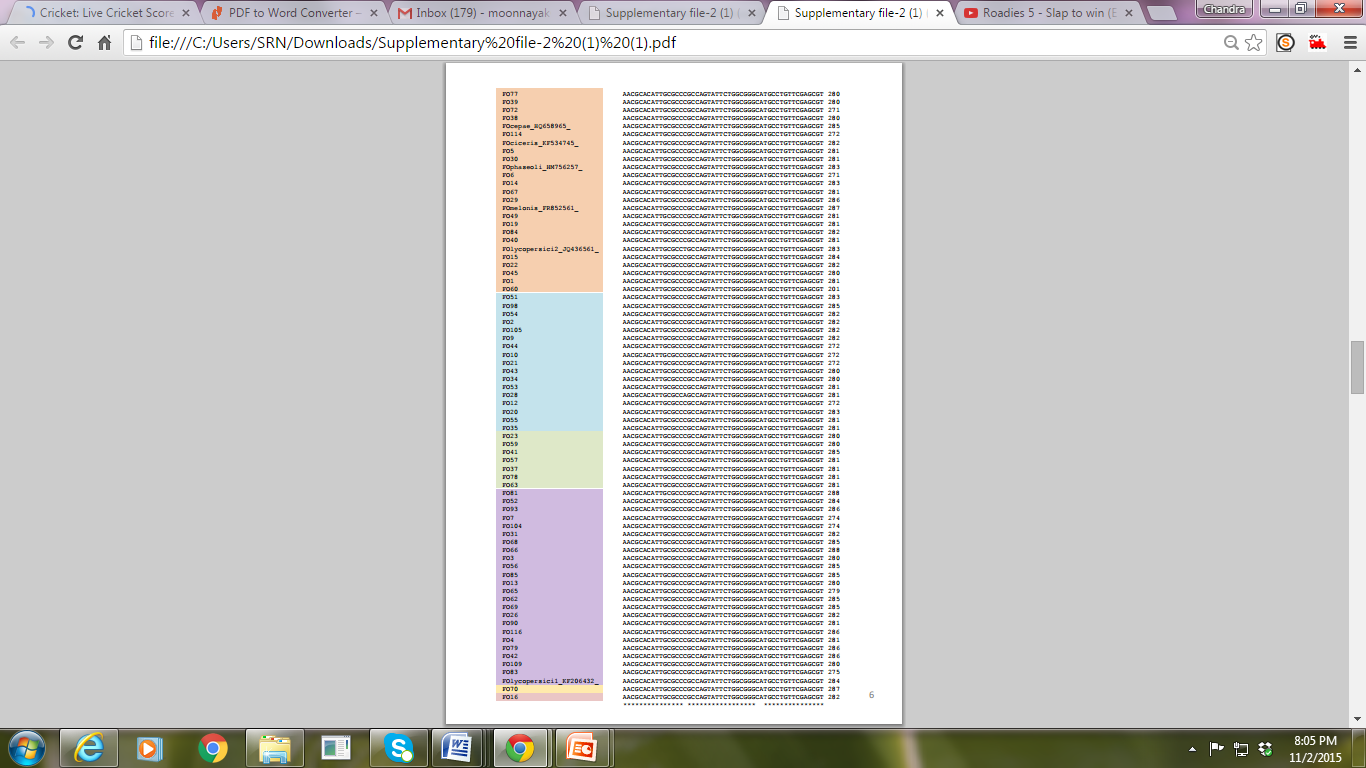


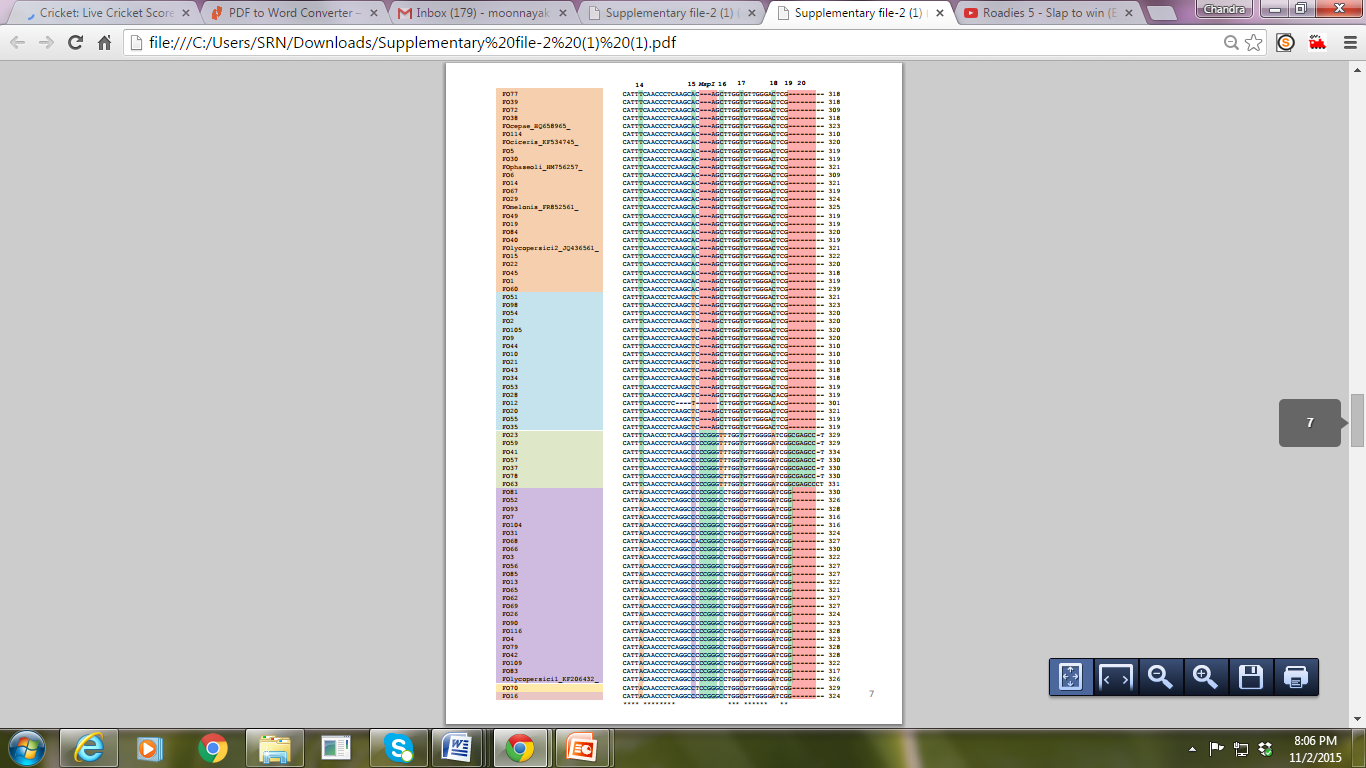


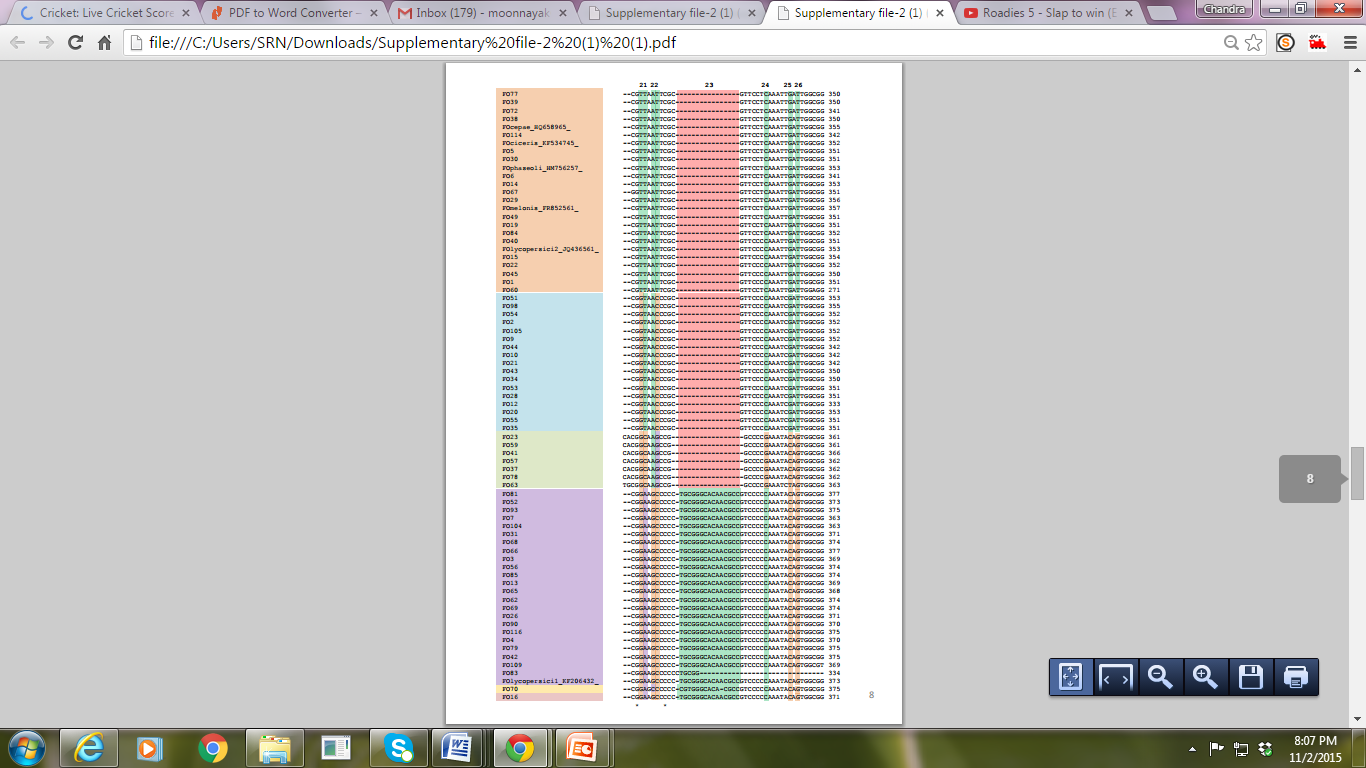


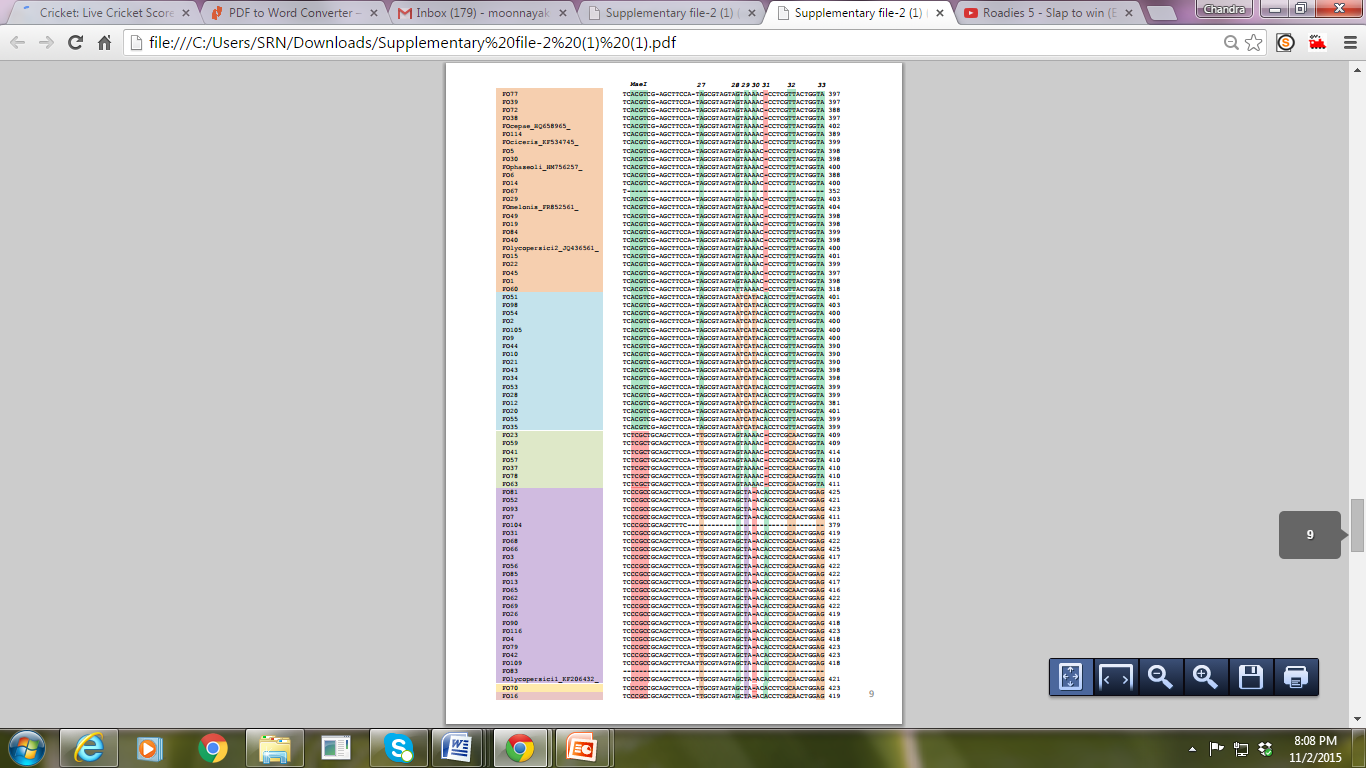


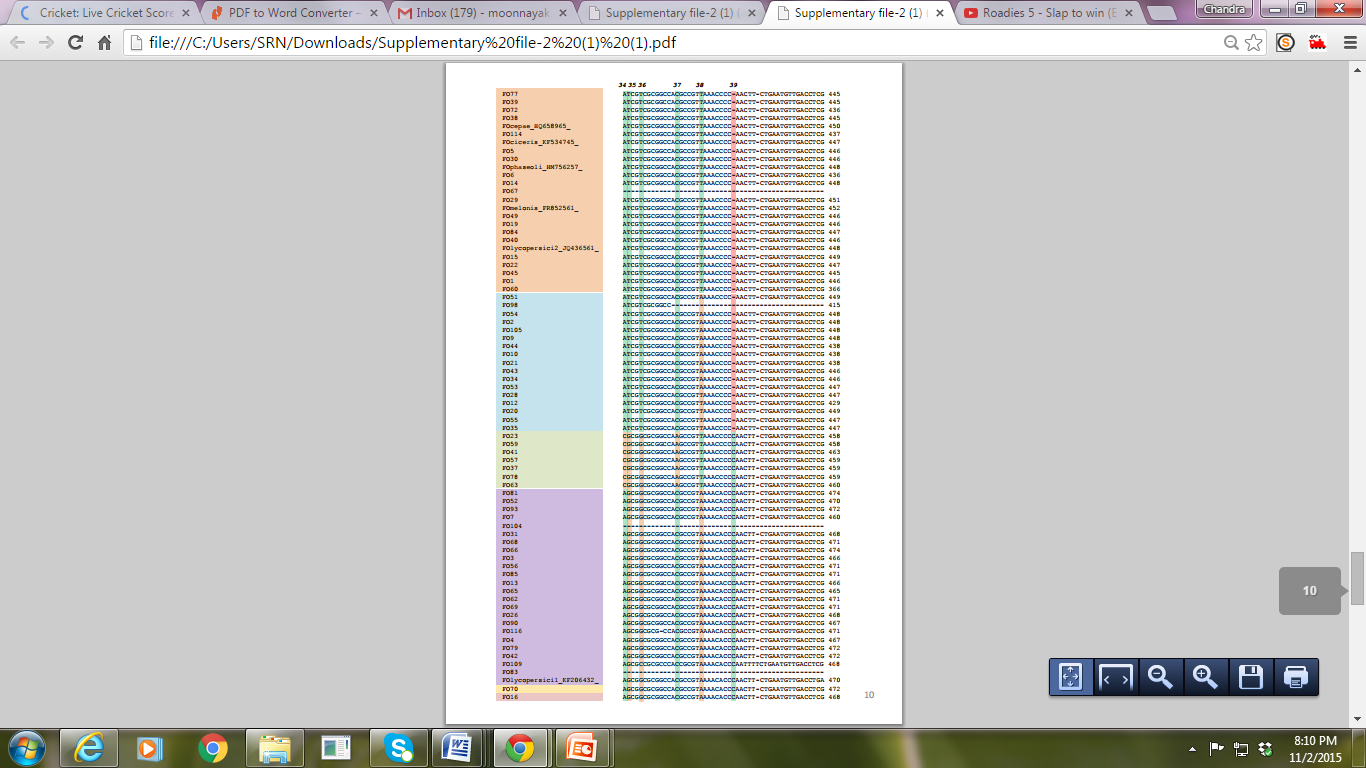


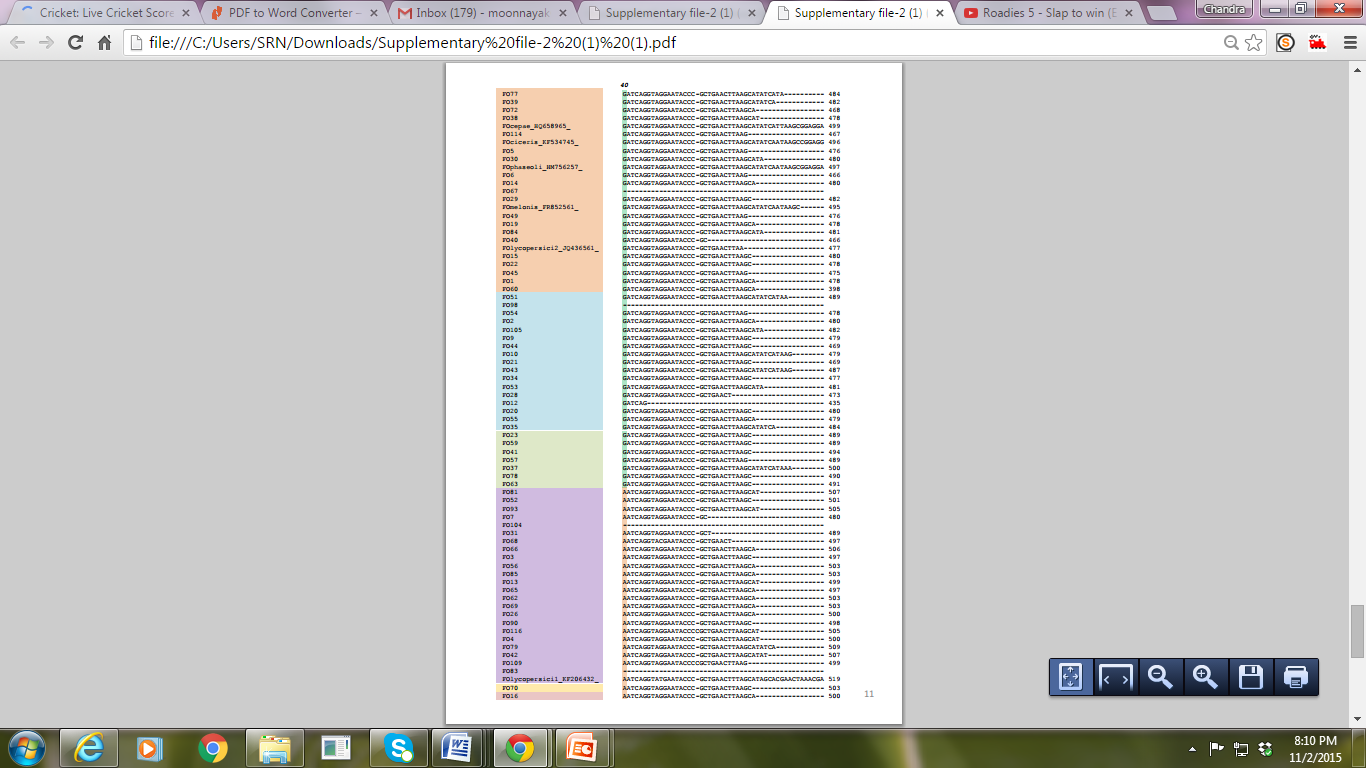


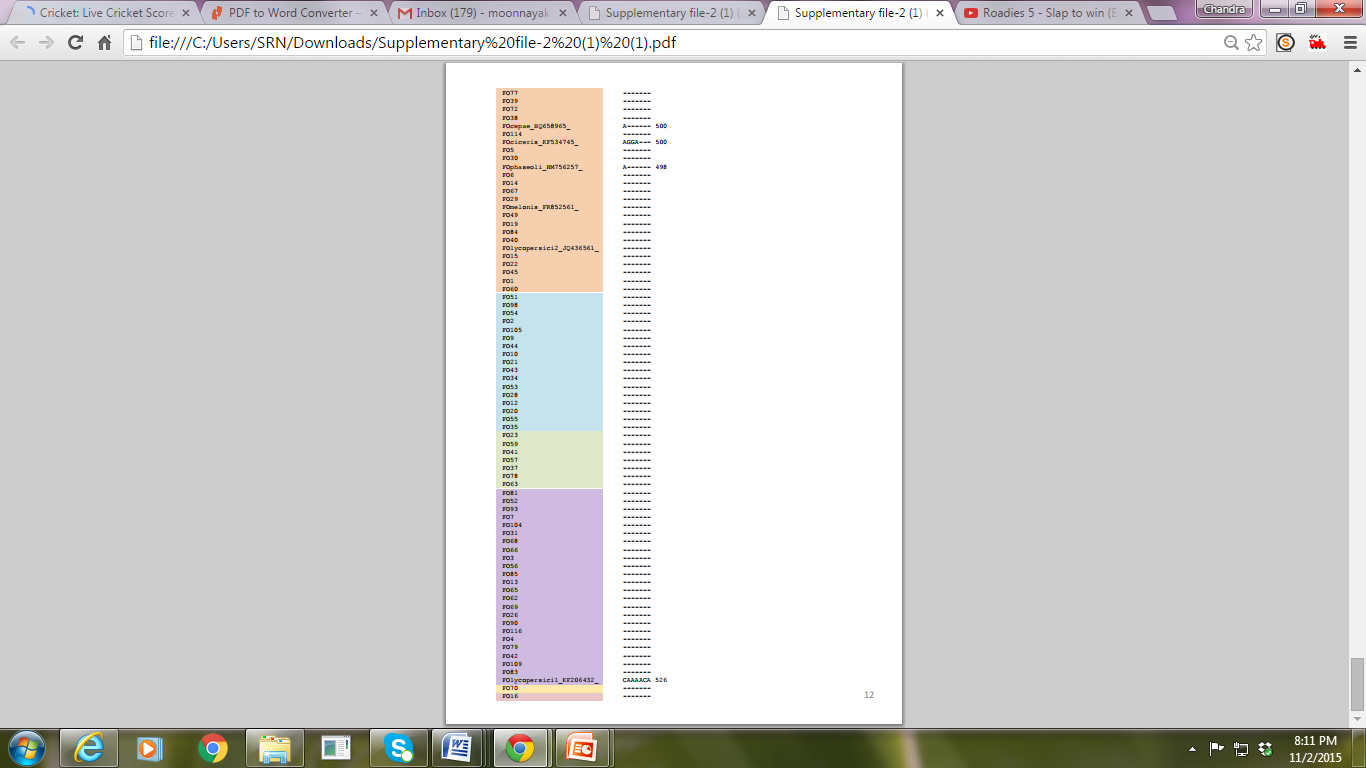


**Figure.2:** The Heatmap profile and diversity distribution


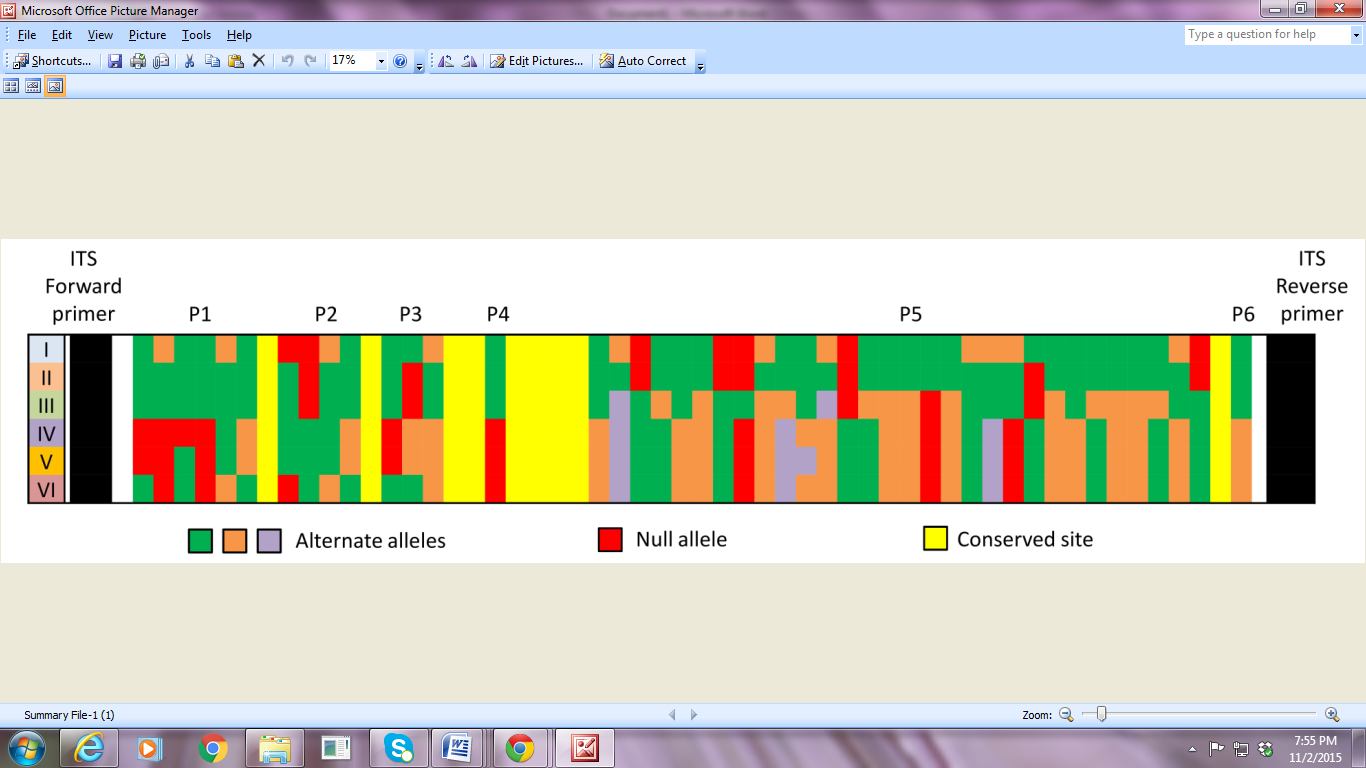

Supplement: Supplementary Information [file srep21367-s1.doc]
